# Cyclodextrin Inclusion Complexes for Improved Drug Bioavailability and Activity: Synthetic and Analytical Aspects

**DOI:** 10.3390/pharmaceutics15092345

**Published:** 2023-09-19

**Authors:** Álvaro Sarabia-Vallejo, María del Mar Caja, Ana I. Olives, M. Antonia Martín, J. Carlos Menéndez

**Affiliations:** 1 Unidad de Química Orgánica y Farmacéutica, Departamento de Química en Ciencias Farmacéuticas, Facultad de Farmacia, Universidad Complutense, 28040 Madrid, Spain; alsarabi@ucm.es; 2 Unidad de Química Analítica, Departamento de Química en Ciencias Farmacéuticas, Facultad de Farmacia, Universidad Complutense, 28040 Madrid, Spain; mcaja01@ucm.es

**Keywords:** drug–cyclodextrin inclusion complexes, nanosponges, cyclodextrin nanofibers, bioavailability enhancement, cyclodextrin synthesis, cyclodextrin analytical techniques, anticancer drug carriers

## Abstract

Many active pharmaceutical ingredients show low oral bioavailability due to factors such as poor solubility and physical and chemical instability. The formation of inclusion complexes with cyclodextrins, as well as cyclodextrin-based polymers, nanosponges, and nanofibers, is a valuable tool to improve the oral bioavailability of many drugs. The microencapsulation process modifies key properties of the included drugs including volatility, dissolution rate, bioavailability, and bioactivity. In this context, we present relevant examples of the stabilization of labile drugs through the encapsulation in cyclodextrins. The formation of inclusion complexes with drugs belonging to class IV in the biopharmaceutical classification system as an effective solution to increase their bioavailability is also discussed. The stabilization and improvement in nutraceuticals used as food supplements, which often have low intestinal absorption due to their poor solubility, is also considered. Cyclodextrin-based nanofibers, which are polymer-free and can be generated using environmentally friendly technologies, lead to dramatic bioavailability enhancements. The synthesis of chemically modified cyclodextrins, polymers, and nanosponges based on cyclodextrins is discussed. Analytical techniques that allow the characterization and verification of the formation of true inclusion complexes are also considered, taking into account the differences in the procedures for the formation of inclusion complexes in solution and in the solid state.

## 1. Introduction

**Figure 1:**
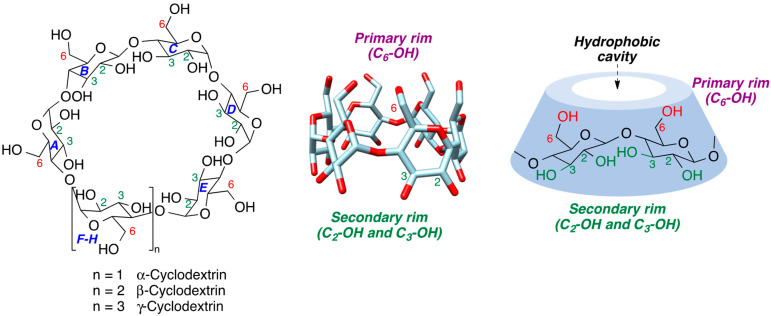
General structure of the natural cyclodextrins. The glucopyranose rings are identified with the letters A–H. Positions 2, 3, and 6 bear hydroxyl groups. The secondary hydroxyls (C_2_ and C_3_) are oriented toward the broader rim of the cyclodextrin cavity and the primary hydroxyls (C_6_) are oriented toward the narrower rim.

Cyclodextrins (CDs) are cyclic oligosaccharides bearing several glucopyranose residues connected by α-1,4 glycosidic bonds. Natural cyclodextrins contain six, seven, or eight glucopyranose units (α-, β- and γ-cyclodextrin, respectively) and are natural products, biodegradable, and generally lacking in toxicity. Cyclodextrins have toroidal shapes, with the smaller opening of the toroid (primary rim) corresponding to the C_6_-OH primary hydroxyls and the larger opening (secondary rim) to the C_2_-OH and C_3_-OH secondary hydroxyls. The inner part of the toroid provides a hydrophobic cavity that can include organic molecules (Figure 1 and Table 1). Thus, the physical and chemical properties of cyclodextrins [1,2,3] make them natural cavitands that have attracted an extraordinary amount of attention in analytical chemistry, organic synthesis and catalysis, and drug delivery, with a large number of applications in the pharmaceutical and food industries [4,5,6,7,8,9,10]. Moreover, their applications have been extended to the recovery of soil and water owing to their capacity to capture pollutants in their cavities [11,12].

The 1987 Nobel Prize in Chemistry was awarded to discoveries on “molecules with structure-specific interactions of high selectivity”. Since then, the development of these structures and their applications in the field of supramolecular chemistry has grown exponentially in all areas, and the development and use of cyclodextrins as drug nanocarriers has become particularly significant, since one of the challenges facing pharmaceutical formulation in the 21st century continues to be increasing the solubility of drugs and improving their chemical, thermal, and photochemical stability. We have witnessed and, at the same time, been involved in the exciting adventure of including pharmaceuticals and nutrients in natural cyclodextrins to improve their organoleptic and biopharmaceutical properties and, later on, the use of chemically modified cyclodextrins custom-designed for the desired vehicle and purpose in a specific pharmaceutical dosage form [13,14,15,16]. In today’s era of personalized medicine, and with therapies that preferably involve oral administration and aim at maintaining therapeutic effects over a prolonged period of time, cyclodextrin-based polymers and specifically nanosponges containing drug–cyclodextrin inclusion complexes [17] constitute a field of research of undoubted importance.

The present review focuses on the improvement in the bioavailability of active pharmaceutical ingredients through the formation of inclusion complexes with cyclodextrins. Under this perspective, the importance of the bioavailability of antitumor drugs is illustrated, as well-established examples of drugs belonging to class IV of the biopharmaceutical classification, owing to the inclusion in the cavities of the CDs, often see their biological activity increased. We have also tried to illustrate the role of cyclodextrins in the resolution of problems related to pediatrics and ophthalmic formulations and to the chemical stability of antibiotics. In this connection, the chemical modifications of the structure of natural cyclodextrins and the simplest and most efficient synthesis routes have been reviewed. Likewise, the synthetic routes for the production of polymers based on cyclodextrins and their inclusion complexes with active principles has also been reviewed. Taking into account that in order to verify the improvement in the bioavailability of drugs it is essential to have sensitive and selective analytical techniques that differentiate the included drugs from the free forms, the analytical techniques used in the characterization of inclusion complexes have been reviewed and summarized in tables, given the high number of articles dealing with this subject.

## 2. Cyclodextrins for Increased Bioavailability

**Figure 2:**
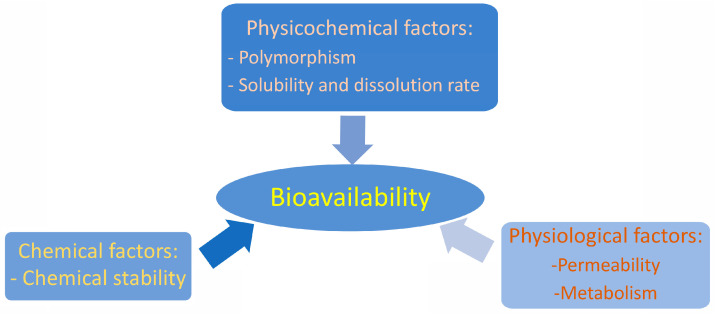
Main factors affecting bioavailability.

The therapeutic efficiency of an active pharmaceutical ingredient depends to a large extent on the excipients accompanying it in the particular pharmaceutical dosage form. Thus, the same quantity of an active substance may achieve a greater, faster or, in other cases, a longer (sustained) effect depending on the excipients accompanying it inside the pharmaceutical dosage form in which it is formulated. Bioavailability can be defined as the fraction of the active substance that, when administered via a route other than intravenous administration, reaches the bloodstream without having undergone transformation. Thus, it is assumed that an active pharmaceutical ingredient administered intravenously has a bioavailability of 100%, but by any other route of administration its bioavailability will be significantly reduced [18] due to many causes that include the physicochemical properties of active pharmaceutical ingredients in the solid state (crystalline and amorphous, polymorphic forms) and their aqueous solubility and dissolution rate in biological fluids. Chemical causes affecting bioavailability include chemical reactivity at physiological pH (hydrolysis, oxidation, metal chelation, and photochemical transformations). Physiological factors affecting bioavailability include the ability to cross cell and tissue membranes as well as structural modifications due to the metabolism of the active substances (Figure 2).

**Figure 3:**
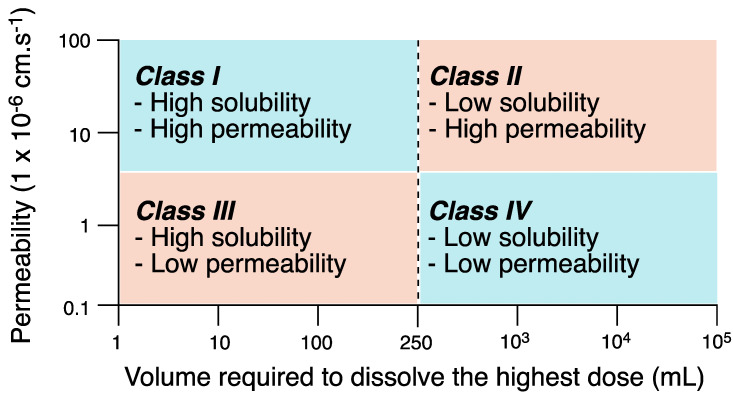
The biopharmaceutical classification system.

The Biopharmaceutical Classification System (BCS), established in the 1990s [19,20], classifies drugs into four groups: Class I, II, III, and IV, differentiating them by their solubility and membrane penetration capacity (Figure 3). The BCS can be used with predictive value for in vivo and in vitro assays, and good extrapolations are obtained by relating drug aqueous solubility to permeability across the gastrointestinal membrane [21,22].

**Figure 4:**
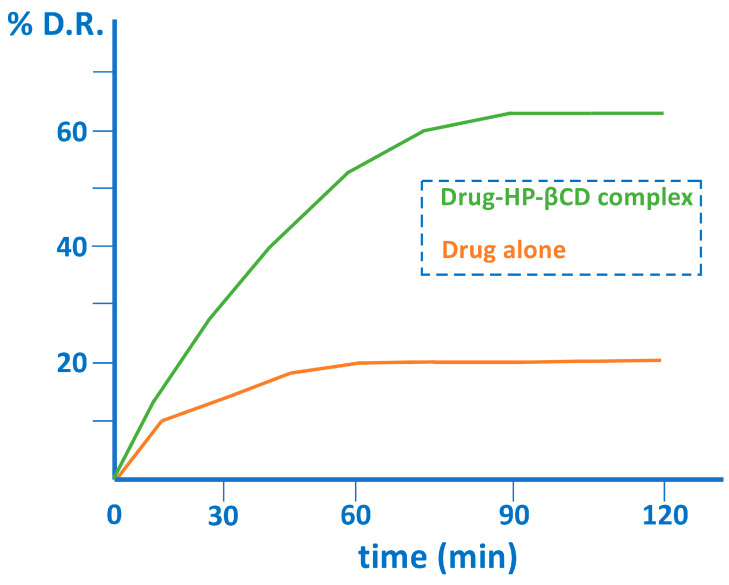
A general illustration of the comparison of the percentage of drug release (% D.R.) in oral formulations. Enhancement of drug release for a drug-CD inclusion complex with respect to the free drug.

The formation of inclusion complexes between drugs and natural or modified cyclodextrins increases aqueous solubility and improves the bioavailability of drugs [23,24,25,26]. On the other hand, cyclodextrin polymers [27] and protein-encapsulated cyclodextrins provide considerable improvements in bioavailability by facilitating membrane penetration and chemical stability [28]. The increase in therapeutic efficacy, in many cases, is a consequence of a modification of the pharmacokinetics of the active pharmaceutical ingredients by increasing the percentage of drug release [29,30,31] and also involves achieving a sustained concentration over time (Figure 4).

**Figure 5:**
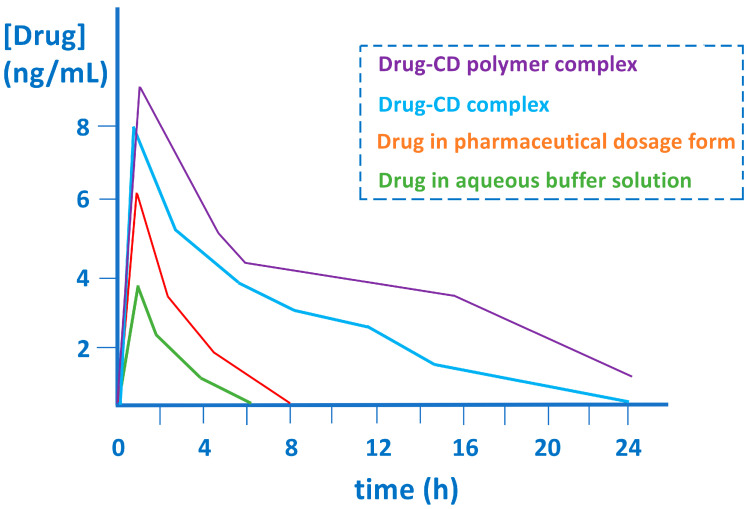
General illustration of the comparison of plasma concentration–time profiles of drugs after oral administration of an aqueous drug solution, commercially available oral pharmaceutical dosage form, and the corresponding CD inclusion complexes and polymers based on CD complexes.

Similarly, the increase in the half-life of the active pharmaceutical ingredients to improve their distribution and therapeutic activity is shown in Figure 5. Thus, the formation of complexes with CDs (natural or chemically modified) or with polymers [27] based on CDs increases the plasma concentration of the active pharmaceutical ingredients [32,33] and also increases the percentage of released drug with respect to the corresponding free drugs. Controlled drug release, as well as improved pharmacokinetic properties and bioavailability can be achieved using CD-based nanomaterials [34] (nanosponges, polymeric nanoparticles, graphene nanoparticles, and silica nanoparticles). These nanomaterials achieve an increase in the stability of many active pharmaceutical ingredients. These advantageous properties must be examined in relation to the chemical characteristics of these nanomaterials because although cyclodextrins are biodegradable and show low toxicity [6], some of these nanomaterials do not have these advantages.

## 3. Cyclodextrins for Improved Drug Solubility

The increased solubility [26,35] of drugs following their encapsulation in the cavities of CDs has gained them inclusion in pharmacopoeias as excipients and adjuvants. By increasing the solubility of class IV (BCS) bioactive compounds, significant positive changes in the bioavailability of drugs are achieved.

**Figure 6:**
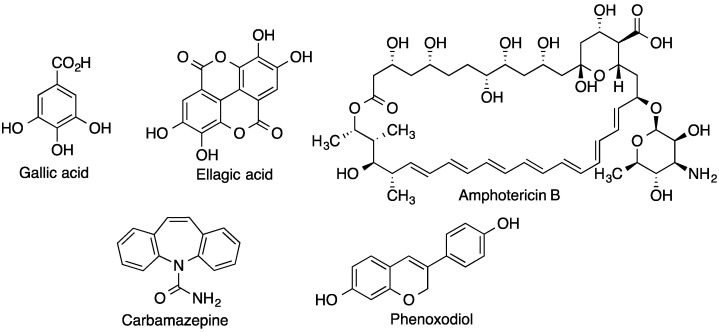
Some compounds whose bioavailability is increased by cyclodextrin inclusion due to improved solubility.

Gallic acid (Figure 6), a natural polyphenol used in traditional Chinese medicine, has interesting antibacterial properties. The formation of inclusion complexes with HPβ-CD makes it possible to increase its activity against Gram-negative bacteria owing to an improvement in solubility of more than 100 times with respect to the drug alone [36].

Ellagic acid, which can be viewed as a dimer of gallic acid (Figure 6), is another polyphenol present in many vegetables that has relevant antioxidant and anti-inflammatory properties, acting as a hepatoprotective agent. It has a planar structure with four rings, which makes it highly lipophilic, and it is also unstable due to the presence of two lactone moieties. For these reasons it has low water solubility and very low oral bioavailability. The formulation of this active pharmaceutical ingredient in β-CD-based nanosponges with dimethyl carbonate as the cross-linking agent has been proposed. This delivery system not only improves the water solubility but also enables the controlled and sustained release of the active ingredient over 24 h [37].

Fungal infections often require local, topical treatment to allow transdermal penetration of antifungal agents. Amphotericin B (Figure 6) is a broad-spectrum antifungal drug, which due to its chemical structure has an amphiphilic character, hence its name. Amphotericin B has low water solubility and therefore very poor bioavailability. The formation of inclusion complexes with CDs that interpose in a stable oil emulsion [38] has led to a significant increase in solubility with encouraging results in its pharmacokinetics and bioavailability.

Another example of a very widely used active substance with low water solubility is carbamazepine (Figure 6), which is used in the treatment of certain neurological disorders such as epilepsy and neuropathic pain. These conditions affect a significant percentage of the population, and therefore problems related to its poor bioavailability due to its slow dissolution remain a challenge. A significant improvement in both the percentage of active ingredient released and the plasma concentration of carbamazepine was achieved by means of hydroxypropyl-β-cyclodextrin (HP-βCD) inclusion complexes and is clearly dependent on the way in which the inclusion complexes were obtained [39].

The case of anti-cancer drugs is also relevant, as most of the cytotoxic compounds have very poor solubility and also poor penetrability in aqueous fluids, with most of them falling into BCS class IV, while also showing toxic effects even at low concentrations. Therefore, the role of natural, modified cyclodextrins and cyclodextrin-based polymers in their administration has been the subject of much research [40,41,42,43]. Moreover, many anti-tumor drugs are highly lipophilic and, therefore, even when administered intravenously, it is sometimes difficult to achieve the concentration required to achieve the desired effect. Phenoxodiol (Figure 6) is an anti-tumor flavone derivative whose activity is significantly reduced due to its poor water solubility [44]. The formation of inclusion complexes with β-CD increases its cytotoxic activity against breast cancer and neuroblastoma cell lines and it also reduces its side effects more than five-fold.

## 4. Cyclodextrins for Improved Drug Stability

In some cases, drugs have stability problems associated with their chemical structure that can be alleviated by cyclodextrin inclusion. Thus, the compartmentalization of isolated molecules in the cavities of cyclodextrins improves their stability by preventing the formation of dimers or protecting against hydrolysis under physiological pH conditions and, in some cases, increases bioavailability by slowing down metabolization. These effects have been reviewed [45] and the effects on the stability of penicillin antibiotics [46], the above-mentioned antifungal compound amphotericin B [38,47], and antidepressant drugs [48] have also been described.

**Figure 7:**
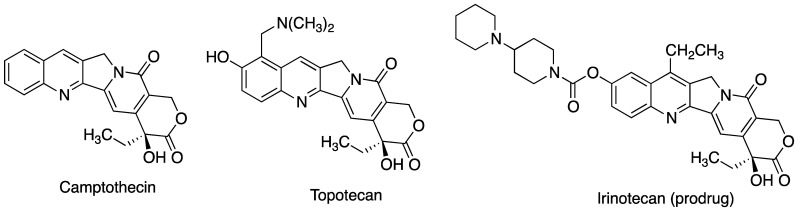
Representative members of the camptothecin family of anticancer drugs.

One particularly challenging case was that of camptothecin (Figure 7), an anticancer topoisomerase inhibitor characterized not only by its lipophilicity but also its low chemical stability, due to the presence of a lactone ring in its structure that is readily hydrolyzed at physiological pH. The stabilization of camptothecin within cyclodextrin cavities reduces the hydrolysis of the lactone ring of camptothecin from 40% to 3.4% in the case of β-CD complexes and to 6.7% in the case of HP-β-CD, leading to a significant increase in antitumor activity in several tumor cell lines [49]. Alternatively, the formulation of the active substance in the form of nanocapsules with chemically modified cyclodextrins (heptakis(6-*O*-hexanoyl)cyclomaltoheptose) and coated with a cationic polymer based on chitosan has been shown to increase the stability of camptothecin and its in vitro effectiveness [50], and a clear enhancement in the stability of camptothecin was shown when camptothecin was formulated as cyclodextrin-based polymeric nanoparticles prepared from poly(lactide-co-glycolide) and poly-ε-caprolactone as biocompatible polymers [51].

Topotecan (TPT, Figure 7) is a synthetic analogue of camptothecin designed to improve its aqueous solubility and widely used for treating refractory lung cancer. As in the case of camptothecin, its active form is a lactone that is hydrolyzed in physiological fluids, resulting in a low bioavailability. TPT-loaded crosslinked cyclodextrin metal–organic frameworks have been shown to protect topotecan from hydrolysis and improve its bioavailability [52].

Irinotecan (Figure 7) is another member of the camptothecin family, acting as a carbamate prodrug, whose stabilization by the formation of inclusion complexes with α-, β-, and *γ*-CD has been studied, leading to the conclusion that stability improvement is greater in the case of β-CD complexes, which show a higher loading capacity [53].

## 5. Cyclodextrins for Solving Formulation Problems

Additional challenges that the use of CDs or their polymeric derivatives has been able to solve are linked to the poor bioavailability of some active pharmaceutical ingredients in certain pharmaceutical dosage forms, such as pediatric and ophthalmic preparations.

**Figure 8:**
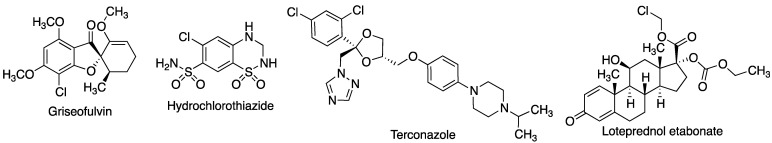
Drugs for which specific cyclodextrin-related formulations have overcome administration issues.

Pediatric formulations are conditioned by multiple factors due to the peculiarities of the target population segment. These factors include the weight of the infant/child, which changes constantly during the first years of life, and the difficulty of ingesting oral pharmaceutical forms frequently used by the adult population. Most commonly, pediatric formulations are extemporaneously prepared liquid presentations, which allow for the correct dosage of active pharmaceutical ingredients according to the weight of the child. Many of the active pharmaceutical ingredients are not water-soluble as required for pediatric oral liquid presentations and therefore the formation of inclusion complexes with cyclodextrins solves one of the problems posed by these presentations. A four- to six-fold improvement in the bioavailability of a pediatric formulation of the antifungal drug griseofulvin [54] (Figure 8) has been demonstrated following its incorporation into β-CD- and β-CD-based nanosponges. This improvement in bioavailability is a consequence of the increase in solubility with respect to the active substance.

Hydrochlorothiazide (Figure 8) shows very low water solubility (class IV) which also poses problems for pediatric formulations. Sulfobutyl-ether-β-CD and HPβ-CD inclusion complexes of this drug have been formulated and incorporated into nanoparticles of surfactant-containing solid lipid dispersed systems. The bioavailability after 120 min is shown to be improved by 3–4 times. Importantly, this improvement in bioavailability over the commercial hydrochlorothiazide suspension is significantly greater for the HPβ-CD than for the sulfobutyl-ether-β-CD nanoparticles, for which little increase in bioavailability is obtained [55].

The ocular mucosa has unique characteristics as it is a barrier to prevent the passage of micro-organisms and other elements and at the same time is a good route for drug administration. The drugs have to cross several lipophilic barriers separated by hydrophilic fluids, and cyclodextrins provide the right hydrophilicity–lipophilicity balance to overcome these barriers [56,57]. Among the infectious pathologies affecting the ophthalmic mucosa, keratitis of fungal and bacterial origin is a serious problem that can become chronic. Azoles, such as terconazole (Figure 8), are highly effective topical antifungals that are widely employed in the treatment of ocular infections due to their good activity and low toxicity. However, their poor solubility is a drawback toward maintaining a prolonged and sustained antifungal action. The formulation of terconazole in silica/chitosan nanoparticles, using CDs as carriers, enables a sustained release of this drug. Thus, the use of dimethyl β-CD (DMβ-CD) as a carrier increases the drug concentration in the ocular mucosa by 5–7-fold compared with the terconazole suspension, thus achieving a significant improvement in its bioavailability [58].

After ophthalmic surgery, corticosteroids are often required to reduce the inflammation associated with the surgical process. Loteprednol etabonate (Figure 8) is a glycocorticoid that displays 1.5–2 times the anti-inflammatory activity of dexamethasone. It also has fewer side effects than classic glycocorticoids and it is therefore frequently used for the treatment of allergy-related inflammatory problems. One of the advantages of loteprednol treatment is that it can be prolonged for more than a month without intraocular pressure problems. The formation of inclusion complexes with β-CD and HPβ-CD and the additional incorporation of hydroxypropyl methylcellulose, methylcellulose, and sodium alginate as gel-forming agents improves the solubility and, therefore, the bioavailability of this active pharmaceutical ingredient. This enhancement is even greater in the case of inclusion complexes with HPβ-CD [59].

## 6. Cyclodextrins for Targeted Drug Delivery

Cyclodextrins and related polymeric nanostructures have found broad application in the selective delivery of drug molecules to specific tissues [60].

### 6.1. Introduction: Cyclodextrin-Derived Nanoparticles and the EPR Effect

**Figure 9:**
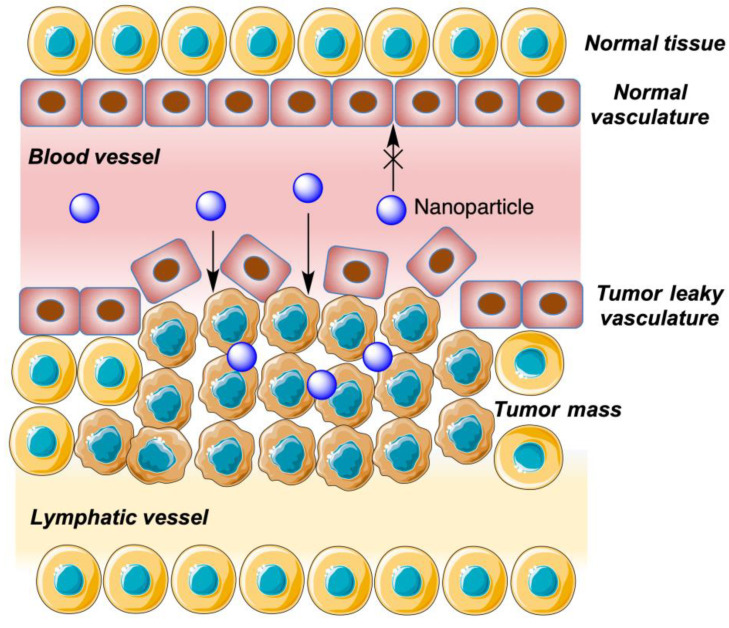
The Enhanced Permeability Retention (EPR) effect. The arrows represent drug movement across the membrane.

The use of cyclodextrin inclusion complexes encapsulated/embedded in liposome/niosome-like entities allows for targeted delivery. On the other hand, polymers based on CDs and polyethylene glycol (PEG), which are non-toxic and display a low antigenic capacity, improve bioavailability by increasing the solubility and chemical stability of some drugs. These nanostructures take advantage of the EPR (Enhanced Permeability Retention) effect, which is related to the fact that certain molecules and macromolecules with a particular size (nanoparticles, nanosponges, liposomes, niosomes, as well as drugs and prodrugs linked to polymers and antibodies) tend to accumulate in tissues with high vascular permeability. Cancerous tissues with increased metabolism and high inflammation are characterized by high vascular permeability and insufficient lymphatic clearance due to the accumulation of a large amount of debris cells. In addition, in many cases the lymphatic vessels are unable to fulfil their mission due to the pressure exerted by the tumor mass. Due to the insufficient work of the lymphatic vessels, there is an accumulation of this type of supramolecular entity in the tumor tissues, effectively contributing to enhance the activity of antitumor drugs in this mode of delivery (Figure 9) [40,42,61].

The use of CDs as components of these drug nano-carriers has the additional advantage of facilitating selective tumor uptake due to the high consumption of glucose by the neoplastic cells.

Paclitaxel is often used in the treatment of metastatic breast cancer, but its oral bioavailability is very low and it also presents problems in its formulation for endovenous administration due to its low water solubility. Therefore, different ways to increase its bioavailability have been tested. Inclusion in native β-CD, HPβ-CD, and aminoβ-CD, followed by the formation of nanoparticles with poly(anhydride), results in improved oral bioavailability. However, significant variations in pharmacokinetics and bioavailability are observed depending on the CD chemistry, with the best results being obtained with HPβ-CD which produces plasma concentrations of paclitaxel four times higher than those obtained with aminoβ-CD [62].

Irinotecan CD complexes were functionalized through conjugation with nanoparticles based on iron oxides and graphene, a strategy that significantly increases the biological activity in human colon adenocarcinoma cells and improves the stability of the active substance [54].

### 6.2. Cyclodextrin-Based Nanoparticles for Combined Photothermal/Chemotherapy

**Figure 10:**
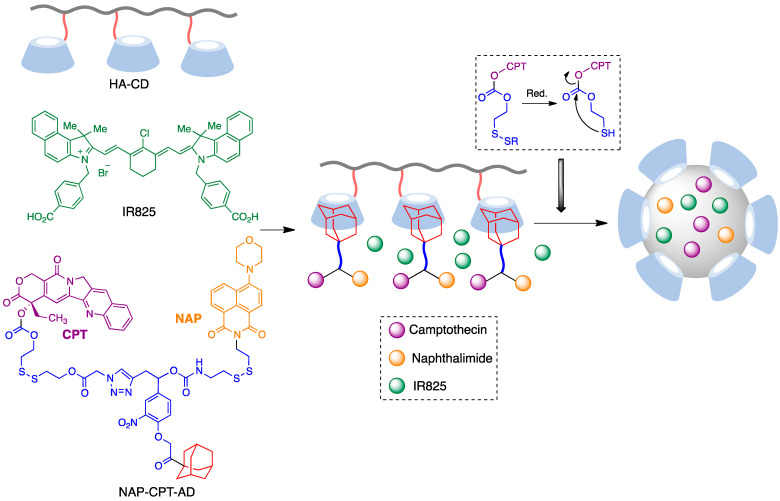
Cyclodextrin-based nanoparticles for combined photothermal therapy/chemotherapy.

CD-pendent polymers can serve as hosts to encapsulate a large number of drug molecules that cannot be readily included by free cyclodextrins. In one example of the application of this strategy, polymeric nanoparticles were prepared by a host–guest interaction between a prodrug of the anticancer drugs camptothecin (CPT) and naphthalimide (NAP) containing an adamantane molecule (AD) and a CD-pendent hyaluronic acid (HA) polymer, which endows the assembly with colloidal stability and biocompatibility. The IR825 photothermal agent, a near-infrared-absorbing dye able to transfer the absorbed light into local heat, was also loaded into the hydrophobic core during the self-assembly process. After entering cancer cells, the disulfide bond in the prodrug is cleaved, leading to the release of CPT and naphthalimide via an intramolecular nucleophilic substitution (Figure 10). Besides the anticancer activity of camptothecin and naphthalimide, upon 808 nm laser irradiation, these nanoparticles absorbed light and transferred the energy to heat efficiently, leading to a temperature of 53 °C at the surface of tumors [63].

## 7. Cyclodextrins for Increasing the Bioavailability of Diet Components

This section will consider some essential compounds that are present in foodstuffs and for which the inclusion in the cavities of CDs represents a considerable improvement from the bioavailability point of view. There is a growing interest in natural compounds (nutraceuticals) used as food supplements with the aim of improving the general state of the organism. Among these compounds, those with antioxidant capacity stand out, due to their potential beneficial effect on cellular/physiological aging. The beneficial effects of cyclodextrins on the bioavailability of various nutraceuticals has been the subject of a great deal of research [64,65].

Many polyphenolic compounds, flavones and isoflavones, as well as terpenes, have beneficial pharmacological properties but have very low water solubility and therefore low bioavailability. Such is the case of curcumin [66], which is the main component of turmeric and, in addition to its antioxidant properties, has anti-inflammatory and anticancer activities. The formation of curcumin/HPβ-CD inclusion complexes increases the plasma concentration of curcumin by two-fold, thus improving its bioavailability compared with free curcumin. In addition, the inclusion complexes show more sustained plasma levels over time and, consequently, the beneficial effects of curcumin are prolonged.

**Figure 11:**
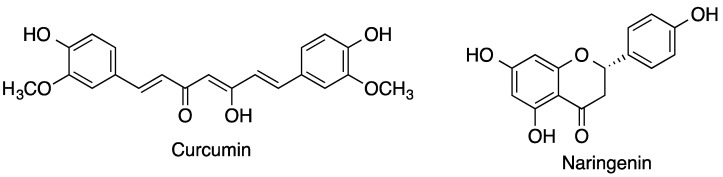
Structures of curcumin and naringenin—two diet components that increase their bioavailability upon CD complexation.

Another interesting example of a compound that is present in many fruits is naringenin (Figure 11). Naringenin is a flavonoid with antioxidant activity and also has hypolipidemic and hypoglycemic activity. Naringenin [67] has very low water solubility, and thus its beneficial effects are limited by its poor bioavailability. The formation of naringenin/HPβ-CD inclusion complexes increases its water solubility by hundreds of times and increases its penetrability 11-fold in the Caco-2 cell membrane transport model. The naringenin/HPβ-CD inclusion complexes also lower plasma glucose levels due to a two-fold increase in glucose clearance compared with free naringenin.

Some additional examples of increased bioavailability via CD inclusion are summarized in Table 2.

## 8. Cyclodextrin Nanofibers

CDs are capable of self-assembling into nanotube-like structures due to intermolecular interactions through hydrogen bonding between the primary and secondary hydroxyl groups on the rims of CD units, connecting each CD unit with the next one. This phenomenon can take place both in solution and in the solid state. CD nanofibers can be produced from either native CDs or chemically modified CDs and have important advantages over CD-based polymeric materials. Thus, CD nanofibers are free of other chemical additives and only CDs are involved, leading to low toxicity in comparison with CD-based polymers. Furthermore, nanofibers based on CDs present high specific surface (surface area to volume ratio) and thus facilitate the solubility and dispersion of many active pharmaceutical ingredients. Nanofibers also present low density and high porosity compared with other conventional fibers [92].

One of the problems associated with the formation of nanofibers is the difficulty in obtaining reproducible degrees of porosity, morphology, and size (diameter and length of the filaments). A successful and reproducible way to achieve the formation of nanofibers that maintain the desired porosity characteristics and dimensions is electrospinning, which is based on the application of a high-voltage electrostatic field to a homogeneous system (solution) or a heterogeneous system (liquid or solid dispersion). The electric field generates a charged jet and charged droplets which are continuously ejected toward a collector device. In this process, solvent is eliminated and thus a solidified nanofiber is originated on the plate surface [93]. In the specific case of nanofibers involving CDs, as well as their inclusion complexes, the energy provided by electrospinning [94] is sufficient to effectively establish the interactions between the CD units and, at the same time, to maintain and elongate the nanofibers and achieve the formation of stable chains, both in the solid state (melts) and in solution. In this way, nanofibers with highly reproducible characteristics (morphology and size distribution) are achieved under fixed experimental conditions (electrical field, flow rate, and tip to collector distance). The use of this technique in the preparation of polymer-free cyclodextrin-based nanofibers and its application in drug delivery through the formation of inclusion complexes with cyclodextrins have been comprehensively reviewed [95,96]. One additional advantage of the use of electrospinning in the production of nanofibers is that it can be considered a sustainable methodology due to the products and reagents used and the almost zero use of solvents, which, together with the use of cyclodextrins, make nanofibers green materials.

**Figure 12:**
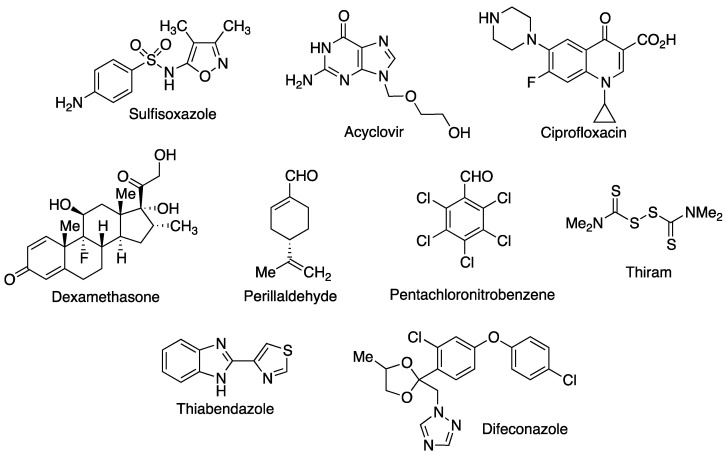
Some drugs and agrochemicals that have been formulated as cyclodextrin nanofibers.

Due to the higher specific surface area of the CD nanofibers, an improvement in aqueous solubility is generally observed with a considerable improvement in the bioavailability of many active pharmaceutical ingredients. Thus, a considerable improvement in drug release concentration is observed when comparing the released concentration of powdered sulfisoxazole (Figure 12) with nanofibers produced by electrospinning of the sulfisoxazole inclusion complex with HPβ-CD and hydroxypropyl cellulose. It is significant that in the case of the CD-based nanofibers, a more sustained action over time was achieved [97].

Acyclovir (Figure 12) is an antiviral widely used in the treatment of the herpes simplex virus, but it has the disadvantage of a very low hydrosolubility and therefore low oral bioavailability. The formation of inclusion complexes with HPβ-CD and the subsequent generation of nanofibers via electrospinning provides an improvement in solubility of the drug for oral administration [98].

Ciprofloxacin and dexamethasone (Figure 12) are a frequent combination of an antibiotic and a steroidal drug employed in the treatment of otitis externa. Both active ingredients belong to class IV (BCS) with low hydrosolubility and permeability. The production of nanofibers based on inclusion complexes with β-CD and polycaprolactone and gelatin as polymeric materials has enhanced drug release and pharmacological activity [99]. Significant improvements in the solubility of hydrocortisone [100] and piroxicam [101] have been reported due to the high degree of wetting of the nanofibers obtained via electrospinning from the inclusion complexes with HPβ-CD, with the advantage of not using additional polymeric materials.

Perillaldehyde (Figure 12) is a natural product with antioxidant and antibacterial activity. Besides being volatile, it has a very low water solubility and therefore very poor bioavailability. The formation of inclusion complexes with HP*γ*-CD and the formation of nanofibers free of other polymeric materials leads to a substantial increase in water solubility. In addition, it has been shown that nanofibers containing the perillaldehyde/HP*γ*-CD inclusion complex have a higher antioxidant capacity, improved thermal stability, and increased antibacterial activity [102].

The prolonged and repeated use of fungicides and other agrochemicals and the fact that some of them have very low water solubility means that they can accumulate both in soils and in the atmosphere. The formation of inclusion complexes with CDs can be applied to remedy these negative effects. It has recently been described that the solubility of pentachloronitrobenzene (Figure 12), an antifungal compound used in agriculture to combat numerous insect pests, is increased by approximately three times owing to the formation of an inclusion complex with HPβ-CD and the formation of nanofibers via electrospinning [103]. The formation of nanofibers based on inclusion complexes of the agrochemical thiram (Figure 12) and HPβ-CD is very effective in the release of this pesticide and conforms to the principles of safety and green and sustainable agriculture [104]. In the search for sustainable formulations, it has been proposed that pesticide inclusion complexes should be formulated to have a higher water solubility and be environmentally friendly. Thus, the formulation of thiabendazole/HPβ-CD inclusion complexes (Figure 12) and the formation of nanofibers free of additional polymers and generated via electrospinning is an attractive approach. The results obtained are successful in the context of green technology and this methodology has been proposed with the aim of reaching a higher level of environmental protection [105]. Similar results have been described for the fungicide difenoconazole (Figure 12) [106].

## 9. Functionalization of Cyclodextrins: Synthetic Aspects

### 9.1. Introduction

The functionalization of natural cyclodextrins is very important for pharmaceutical applications. Although natural CDs are hydrophilic, their aqueous solubility is relatively low due to their tendency to generate aggregates by intermolecular hydrogen bonding. This may cause some nephrotoxicity, especially in the case of β-CD, due to the renal accumulation of insoluble cyclodextrin crystals or cyclodextrin–cholesterol complexes. Thus, much effort has been devoted to the chemical modification of natural cyclodextrins in order to improve their solubility and complexation capacity. Over 11,000 cyclodextrin derivatives have been prepared by incorporation of various moieties such as amines, amino acids, peptides, and aromatic rings. Moreover, more sophisticated supramolecular systems such as polymers, metal–organic frameworks, hydrogels, and other supramolecular assemblies have also been assembled from cyclodextrins [107].

**Figure 13:**
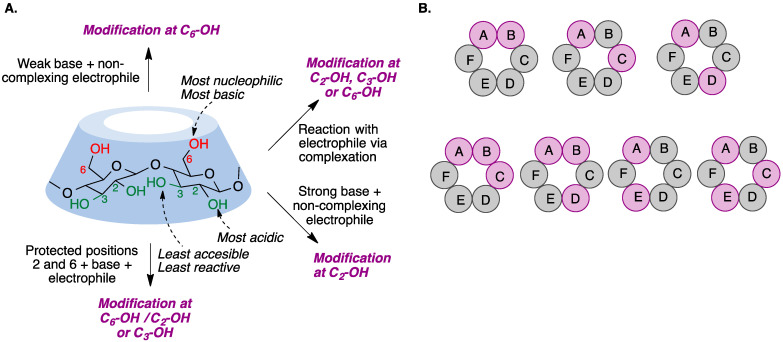
(**A**) Basic functionalization rules for cyclodextrins. (**B**) Positional isomers of difunctionalized (upper row) and trifunctionalized (bottom row) derivatives of α-cyclodextrin. The capital letters represent the individual glucose units.

The chemical modification of cyclodextrins is carried out via reactions in nucleophilic hydroxyl groups. However, selective cyclodextrin functionalization, as opposed to random substitution, is a challenging task because their hydroxyl groups exhibit only slight differences in their reactivity. There are, nevertheless, some characteristic properties for each position that may allow for selective modification if moderately reactive reagents are employed: the hydroxyl group at position 2 is the most acidic, the hydroxyl at position 6 is the most nucleophilic, and the hydroxyl residue at position 3 is the least accessible due to steric hindrance and hydrogen bonding, and hence the least reactive [108]. On the other hand, cyclodextrins can form complexes with reagents in different ways depending on the size of the cavity and the solvent, modifying their reactivity and sometimes making their performance unpredictable [109] (Figure 13A). To add an additional layer of complexity, many different functionalization patterns can be obtained for the cyclodextrins resulting from a functionalization reaction (Figure 13B).

Three main strategies can be employed for the preparation of functionalized cyclodextrins [110]:

(a) Straightforward selective modification of the desired positions. This is chemically challenging due to the competition between the hydroxyl groups at positions 2, 3, and 6 of the glucopyranose.
(b) Protection–deprotection strategies which involve taking advantage of the different reactivity of the hydroxyl groups toward protection and deprotection conditions. Deprotected hydroxyl groups can then undergo the desired modifications.
(c) Non-selective modification of hydroxyl groups to provide a mixture of derivatives that is then purified to isolate the desired one. This method generates a large number of undesired by-products, requires tedious purifications, and is not chemically efficient.

### 9.2. Selective Modification of the Primary Rim

#### 9.2.1. Monosubstitution at C_6_-OH

**Figure 14:**
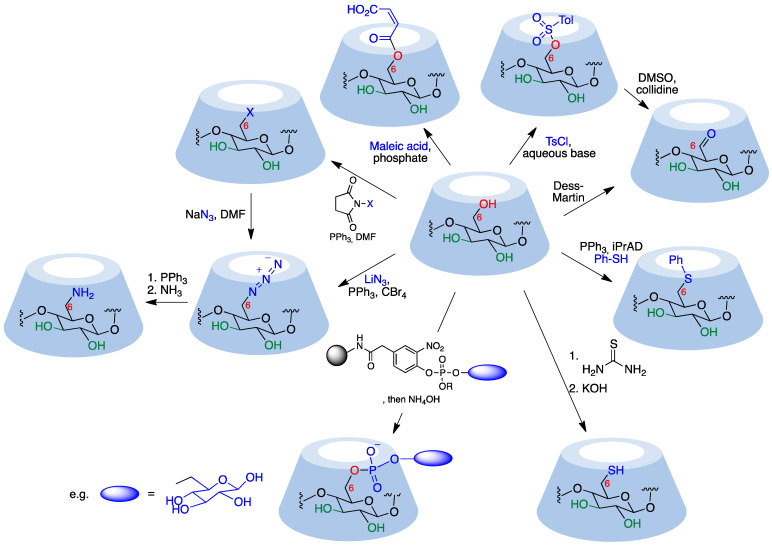
Representative monofunctionalization reactions at the cyclodextrin primary rim.

Primary hydroxyl groups are more nucleophilic than their secondary counterparts, making modification of the primary rim of cyclodextrins relatively straightforward. Electrophiles react with the hydroxyl groups at C-6, yielding the desired product and an acid, which is neutralized by a base added to the reaction medium in order to avoid decomposition of acid-sensitive cyclodextrins. Common strong electrophiles suitable for nucleophilic attack by C_6_-OH include alkyl, sulfonyl, phosphoryl, silyl, and acyl chlorides [64,109]. Some representative transformations are summarized in Figure 14, and will be discussed below.

Monosubstituted *O*-sulfonylcyclodextrins are commonly used as substrates for the attack of nucleophiles to introduce functional groups at position 6. Mono-6-tosylcyclodextrin, for instance, is prepared via the reaction of the starting cyclodextrin with tosyl chloride in an aqueous basic environment [111,112] and can be further attacked by nucleophiles to yield 6-monofunctionalized cyclodextrins after chromatographic purification. Preactivation of the tosylate with imidazole improves selectivity toward 6-O-monotosylation [113]. The reaction with *p*-toluenesulfonic anhydride, on the other hand, does not require purification via chromatography since unreacted anhydride can be simply removed using filtration [114]. Strong bases need to be avoided because they can deprotonate the more acidic hydroxyl group in position 3, generating an alkoxide that displaces the leaving group at C-6 yielding a bicyclic structure.

Aldehydes are versatile groups for further functionalization. α, β, and γ-cyclodextrins can be converted to their mono-6-formyl derivatives in a straightforward manner using the Dess–Martin periodinane reagent [115]. An alternative, indirect way to obtain 6-formylcyclodextrins involves the reaction of the corresponding O-tosyl derivatives with DMSO and collidine [112,116].

The direct introduction of a single aromatic thioether is achieved via a thio-Mitsunobu reaction where β-cyclodextrin is mixed with thiophenol and diisopropyl azodicarboxylate in dimethylacetamide to yield the mono-6-phenylthio derivative with relatively good selectivity over the di- and trisubstituted by-products. The relative amounts of differently functionalized cyclodextrins could be tuned by modification of the reaction conditions [117].

A solid phase method provides mono-6-functionalized α and β-cyclodextrins bearing a variety of complex substituents bridged through a phosphodiester link from the unprotected native cyclodextrins. The Novagel amino solid support was derivatized with 4-hydroxy-3-nitrophenylacetic acid, which was then functionalized with a suitable phosphoramidite derivative containing the structural fragment to be attached to the cyclodextrin (e.g., a saccharide). Cyclodextrins were added to this support in the presence of 1-(mesitylene-2-sulfonyl)-3-nitro-1,2,4-triazole (MSNT) as a coupling reagent, and this was followed by treatment with aqueous ammonia [118].

Mono-6-azidocyclodextrins are accessible through a reaction of α, β, or γ-cyclodextrins with lithium azide, triphenylphosphine, and carbon tetrabromide [119]. However, di- and triazido cyclodextrins are formed as well, making a purification step necessary. Azido cyclodextrins can be readily reduced to the corresponding amino derivatives using the Staudinger reaction [120] or hydride donors [121] and are good 1,3-dipoles for click chemistry.

Together with tosylation, halogenation is the most common starting point for cyclodextrin functionalization. The most convenient and general route to these halo derivatives uses *N*-halosuccinimides as the halogenating reagent [122]. These halides (and also the tosylates) can be readily transformed by nucleophilic substitution. For instance, their reaction with an excess of sodium or lithium azide quantitatively provides an alternative route to mono-6-deoxy-6-azido-cyclodextrins.

Selective mono-6-esterification of β-cyclodextrin has been achieved through its reaction with maleic acid or itaconic acid to selectively yield the corresponding monoesters using phosphate as a catalyst in a semi-dry process [123].

In another approach, selective protection of the secondary rim makes the primary rim readily available for modifications. Taking advantage of their higher basicity, the secondary hydroxyl groups at position 2 of β-cyclodextrins can be selectively protected with *t*-butyldimethylsilyl chloride using NaH as base. The introduction of these bulky groups allows for the primary hydroxyls to undergo reactions with electrophiles without competition from the C-2 or C-3 hydroxyls. The mild removal of silyl protective groups from the secondary rim is then accomplished using tetrabutylammonium fluoride [124].

#### 9.2.2. Disubstitution at C_6_-OH

Compared with monosubstitution, the synthesis of disubstituted cyclodextrins entails an additional issue: the formation of regioisomers, which need to be separated using chromatographic techniques. However, in some cases it is possible to guide the reaction toward the formation of a specific regioisomer.

**Figure 15:**
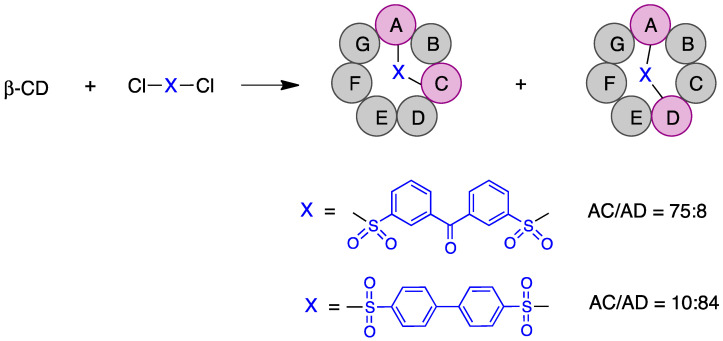
Regioselective AC and AD capping of β-cyclodextrin.

Some of the first cyclodextrin capping strategies involved the introduction of aromatic residues from aromatic disulfonyl chloride reagents. Highly regioselective capping was found in the case of benzophenone-3,3′-disulfonylchloride, which mainly resulted in the AC regioisomer, while biphenyl-4,4′-sulfonyl chloride mainly afforded the AD capping product (Figure 15) [125].

Diiodide derivatives obtained from disulfonyl cyclodextrins can also serve as substrates for further modifications through nucleophilic attack. Sulfonyl groups are displaced via reaction with iodide ion to yield the corresponding diiodo cyclodextrins [126]. Similarly, diazido cyclodextrins can be readily obtained from sulfonyl derivatives [127].

**Figure 16:**
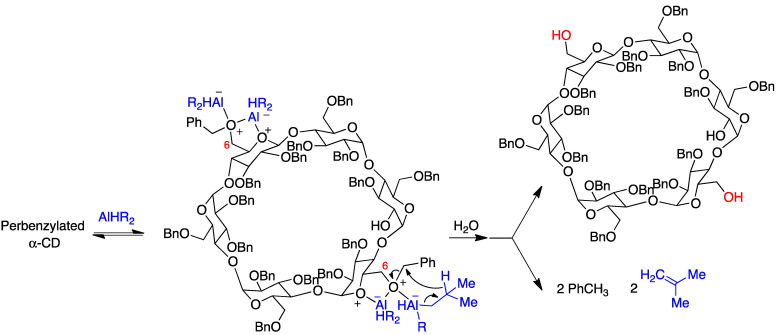
Selective debenzylation of the AD primary hydroxyls in α-cyclodextrin by DIBAL-H.

The selective synthesis of AD diols at the primary side is achieved through a DIBAL-H-mediated regioselective deprotection of the primary hydroxyl groups of perbenzylated α, β, and γ cyclodextrins [128]. For steric reasons, this bis-debenzylation takes place at the opposite glucose rings and involves two independent Lewis acid-assisted debenzylations (Figure 16). It was possible to stop the deprotection after the first debenzylation and isolate the monohydroxy cyclodextrin [129].

Perbenzylated α-cyclodextrins can be functionalized using an iterative process that predictably directs the debenzylation steps to access cyclodextrins with two, three, and five points of attachment for one, two, or three different new functions [130]. In a refinement of the method, through five consecutive site-directed debenzylations mediated by diisobutylaluminium hydride (DIBAL-H), a 2,3-tetradecabenzylated cyclodextrin hexa-hetero-functionalized with six different substituents at the primary hydroxyls was accessed [131].

#### 9.2.3. Other C_6_-OH Substitution Patterns

Trisubstitution of cyclodextrins is a difficult goal for the variable substitution degree the reactions provide as well as for the generation of numerous positional isomers. However, some methods have successfully introduced three substituents in the primary side of cyclodextrins. Thus, complex di- and trifunctionalization patterns on the primary side of α-cyclodextrins are readily accessible owing to selective, iterative deprotections of perbenzylated α-cyclodextrin, where the first steps guide the selectivity of the subsequent ones. These modifications allow to selectively deprotect certain positions and also introduce deoxymethyl and deoxyvinyl residues at C-6 [131]. Similarly, perbenzylated α-cyclodextrins were selectively deprotected to yield a variety of primary-rim-functionalized compounds such as an ABD trihydroxy perbenzylated cyclodextrin and an AD diol, CF O-diTBS-protected perbenzylated cyclodextrin containing three pairs of functionalities on its primary rim [132].

Regarding the complete functionalization of primary alcohols at the primary rim of cyclodextrins, it has, in principle, the advantage that no positional isomers are generated. Nevertheless, factors such as steric crowding and complex formation decrease the yield as the degree of substitution increases. Sulfonylation with tosyl chloride is a good approach to perfunctionalize cyclodextrins, and has been applied to α- [133], β- [134], and γ- [135] cyclodextrins. Persulfonylated cyclodextrins are generally used as intermediates toward other functionalized CD derivatives.

Perhalogenated cyclodextrins also represent a good intermediate for further functionalization, with a higher stability than sulfonyl derivatives but with compromised solubility in non-polar solvents. Direct per-6-iodination of α and β-cyclodextrins can be achieved through reaction with iodine and triphenylphosphine in DMF. Similarly, the reaction of β-cyclodextrin with triphenylphosphine and bromine yields the corresponding per-6-brominated β-cyclodextrin [136]. α, β, and γ-cyclodextrins react with chloromethylenemorpholinium chloride and bromomethylenemorpholinium bromide in DMF to furnish the per-6-chlorinated and per-6-brominated cyclodextrins, respectively [137]. These per-6-halo cyclodextrins are very useful starting materials for additional functionalization processes such as the synthesis of per-amino derivatives and amino acid derivatives [122]. The use of per-6- iodo-per-6-deoxy-β- and γ-cyclodextrins for the synthesis of azido or mercapto derivatives has been performed under solvent-free conditions in a planetary ball mill. This mechanochemical protocol simplified the isolation and purification processes, while also allowing an efficient scale-up [138].

Per-6-alkylation of cyclodextrins is not a straightforward transformation and requires the adoption of a protection–deprotection strategy. An example with α-cyclodextrin involves, in this order, selective protection of the primary hydroxyls as silyl ethers, benzylation of the secondary hydroxyls, selective desilylation of the primary side, reaction of the primary hydroxyls with methyl iodide under strongly basic conditions (sodium hydride), and a final hydrogenolysis of the benzyl ethers [139].

The selective introduction of azido groups at all primary hydroxyls is achieved by the reaction of β-cyclodextrin with triphenylphosphine, lithium azide, and carbon tetrabromide in DMF with further treatment with acetic anhydride to yield heptakis(6-azido-6-deoxy)-β-cyclodextrin-tetradeca(2,3)acetate [140]. Per-azido cyclodextrins have been investigated as substrates for copper-catalyzed azide–alkyne cycloadditions [141].

### 9.3. Selective Modification of the Secondary Rim

In general, chemical modification of this side of cyclodextrins is not as straightforward as at the primary rim. This side is more crowded and hydroxyl groups at positions 2 and 3 establish hydrogen bonds making it more rigid. Furthermore, 2 and 3 hydroxyls differ in their reactivity: while the hydroxyl group C-2 is the most acidic of the glucopyranose and is selectively deprotonated with strong bases such as NaH, the C-3 hydroxyl is the least reactive.

#### 9.3.1. Monosubstitution at C_2_-OH

β-cyclodextrin can be transformed into the corresponding 2-monotosyl-β-cyclodextrin via reaction with *m*-nitrophenyl tosylate [142]. The ability of cyclodextrins to form complexes with chemical reagents through the hydrophobic cavity modifies their reactivity, and this feature is used here to selectively direct the reaction to the secondary rim of the cyclodextrin instead of the more reactive primary rim. This complex-formation-based approach was optimized with the design of a more efficient tosyl donor, 1-(*p*-tolylsulfonyl)-1,2,4-triazole [143]. An additional method to selectively synthesize monotosyl α and β-cyclodextrins involves their reaction with N-tosylimidazol in DMF and molecular sieves [144] or under solid-state conditions, using mechanochemical activation [145]. 2-Monotosyl cyclodextrins are key intermediates for the functionalization of not only C-2 but also the C-3 position through the formation of an epoxide.

Direct alkylation at position 2 of β-cyclodextrin is achieved through its reaction with dialkyl sulfates in dilute aqueous alkali [146]. An indirect method to reach position 2, as well as position 3, is the regioselective bis-O-demethylation of 2,3,6-permethylated α and β-cyclodextrins with DIBAL-H in toluene to yield 2A,3B-diol permethylated cyclodextrins as major products, allowing for selective functionalization of a single 2 hydroxyl and/or a single 3 hydroxyl [147,148].

#### 9.3.2. Disubstitution at C_2_-OH

The reaction in DMF of γ-cyclodextrin with benzophenone-3,3-disulfonylimidazole, a rigid disulfonyl precursor, selectively yields 2AB-disulfonylated γ-cyclodextrin [149]. A related rigid disulfonyl imidazole reagent, 1,4-dibenzoyl benzene-3′,3″-disulfonyl imidazole, selectively orients the sulfonylation toward the positional isomer 2AC in α, β, and γ-cyclodextrins [150]. The design of a longer disulfonyl imidazole bridge yielded the positional isomer 2AD in α, β, and γ-cyclodextrins, with a better selectivity for β [151]. These 2-disulfonyl cyclodextrins can serve as versatile building blocks for further modifications at position 2 as well as position 3 through the formation of an intermediate epoxide or by treatment with ammonia to yield the corresponding amine.

#### 9.3.3. Persubstitution at C_2_-OH

Persubstitution at position 2 is favored by the use of strong bases that deprotonate C_2_-hydroxyls. However, the successive introduction of electrophiles makes the secondary rim increasingly crowded, making subsequent substitutions more difficult and shifting the reactivity toward the primary hydroxyls. To avoid this, per-2-tosylations and per-2-alkylations are often carried out after the protection of primary hydroxyls with silyl groups, which can be deprotected afterward [152]. An exception is the selective per-2-methylation of β-cyclodextrin with methyl iodide, sodium hydride, and DMSO [153], or using N-tosylimidazole and a catalytic amount of Cs_2_CO_3_ in DMF [154].

A different approach for the selective per-2-alkylation of cyclodextrins starts with the selective per-2,6-silylation of β-cyclodextrin with *tert*-butyldimethylsilyl chloride and pyridine. Then, during the reaction with methyl iodide and sodium hydride, the silyl groups at the C-2 hydroxyls migrate to the C_3_-OH groups, and the deprotected position 2 is selectively methylated. A final desilylation affords the per-2-methylated cyclodextrin [155]. This C_2_-OH to C_3_-OH migration is also observed in other cases where a more complex substitution pattern is present [156,157].

#### 9.3.4. Monosubstitution at C_3_-OH

C-3 in cyclodextrins are the least reactive hydroxyls in the glucopyranose ring due to their low accessibility. A common strategy to functionalize position 3 consists of the tosylation at C_2_-OH and treatment with a base to generate an epoxide, followed by the introduction of nucleophiles [158]. This provides a mixture of 2- and 3-substituted products that can be separated with chromatographic techniques, with substitution at position 3 being highly favored in most cases. The combination of *N*-benzoylimidazole and carbonate buffer in DMF gave mainly acylation C-3 hydroxyl groups of *β*-CD, which was ascribed to acylation at both C_2_-OH and C_3_-OH accompanied by C_2_-OH to C_3_-OH acyl migration [159]. In other transformations, reagent complexation in the cyclodextrin cavity may direct the reaction to C_3_-OH. For instance, the 3-sulfonylation of α-cyclodextrins is achieved by direct reaction with β-naphtalenesulfonyl chloride [160]. A particular case of alkylation is the selective introduction of an alkyl group at just one 3 position of β-cyclodextrins through the reaction of per-6-terbutyldimethylsilylated β-cyclodextrin with N-methyl-4-chloromethyl-2-nitroaniline in the presence of lutidine [161].

#### 9.3.5. Di- and Trisubstitution at C_3_-OH

In a similar way to that of monosubstitutions at position 3, the manno-2,3-epoxycyclodextrin is a common intermediate used for the introduction of nucleophiles. The positional isomers obtained depend on the initial 2-ditosylate involved in the route. Also, 3-sulfonylation of α-cyclodextrins is achieved via reaction with β-naphtalenesulfonyl chloride and further isolation of the 3-disulfonylated derivatives, particularly a mixture of positional isomers 3AC and 3AD disulfonyls that are obtained due to an oriented synthesis owing to complex formation between the β-cyclodextrin and sulfonyl chloride [162]. The mixture of regioisomers is easily resolved with reverse-phase column chromatography.

The sulfonylation of β-cyclodextrin with β-naphtalenelsulfonyl chloride in basic aqueous acetonitrile was selectively found to yield the 3ACE-trisulfonylated isomer [163].

#### 9.3.6. Persubstitution at C_3_-OH

Perfunctionalization at position 3 of cyclodextrins is a challenging task because 3-hydroxyls are the least reactive and are involved in hydrogen bonding with 2-hydroxyls. Furthermore, they are located in a crowded side of the cyclodextrin which becomes even more crowded after protection of 2-hydroxyls, hindering further reactions.

Despite all these setbacks, perbenzylated α-cyclodextrin can be selectively deprotected at position 3 with Et_3_SiH and I_2_ to provide a 2A–F,6A–F-dodeca-*O*-benzyl α-cyclodextrin. This strategy works as well starting from a 2A–F,3A-F,6A-E-heptadec-*O*-benzyl α-cyclodextrin and a 2A–F,3A-F,6BCEF-hexadec-*O*-benzyl α-cyclodextrin to yield the corresponding 3-deprotected derivatives with one or two deprotected hydroxyls in the primary side, respectively. These derivatives stand out as useful starting materials for the selective perfunctionalization of the relatively unreactive position 3 [164].

### 9.4. Persubstitution of the Primary and Secondary Rims

Although steric hindrance of persubstituted cyclodextrins make the introduction of substituents increasingly difficult, the absence of regioisomers is a major advantage in the synthesis of 2,3,6-perfunctionalized cyclodextrins. Perbenzylation of α and β-cyclodextrins is performed in excellent yield (>90%) by treatment with benzyl chloride and sodium hydride [130]. Similarly, peralkylation of β-cyclodextrin is achieved in 74% yield via reaction with methyl iodide and sodium hydride in DMSO [165].

### 9.5. Cyclodextrin-Based Polymers

CD-based polymers are the basis for new CD-based materials for different types of application fields for pharmaceutical and biomedical applications, but are also useful in cosmetic and food science [166,167,168].

#### 9.5.1. Radical Polymerization Reactions

**Figure 17:**
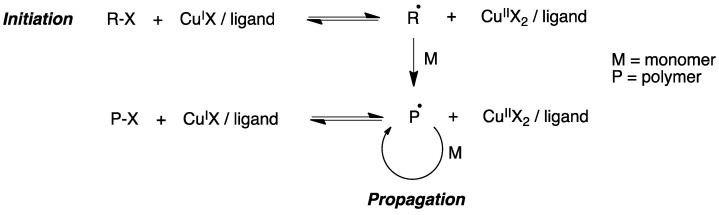
Mechanism of atom transfer radical polymerization (ATRP).

Atom transfer radical polymerization (ATRP) is a type of radical polymerization having as the key step a transition metal-catalyzed C-C bond generation. Generally, an alkyl halide R-X is used as the initiator and a complex formed by a transition metal (e.g., Cu^I^) and a chelating agent is used to generate radicals via a one-electron transfer process, while simultaneously the transition metal is oxidized to a higher oxidation state (Figure 17).

**Figure 18:**
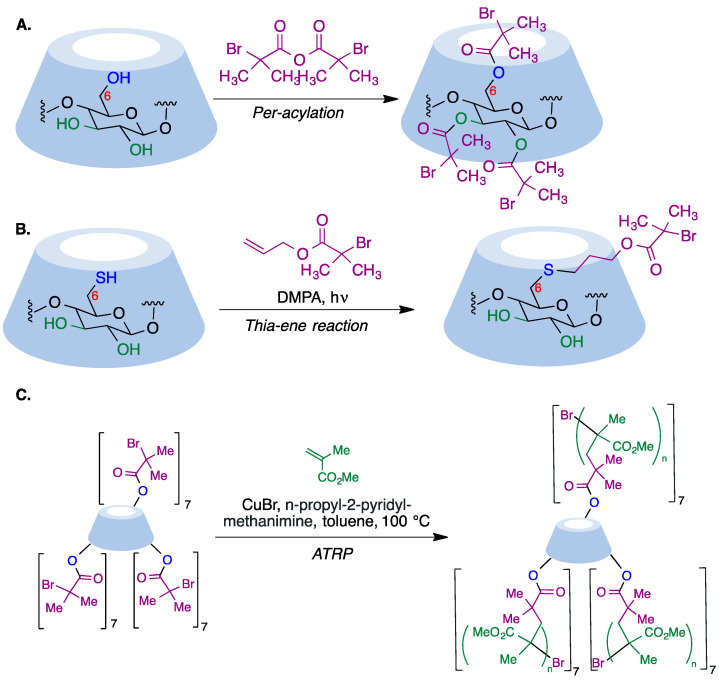
(**A**) Synthesis of per-acylated β-CD via treatment with 2-bromoisobutyryl anhydride. (**B**) Selective functionalization of the cyclodextrin primary rim using a radical thiol–ene reaction. (**C**) Core-based synthesis of a 21-arm star polymer using the ATRP method.

α-Haloester moieties attached to cyclodextrin-based monomers have been widely employed for performing ATRP processes. These moieties can be introduced by direct per-acylation of β-CD with 2-bromoisobutyryl anhydride (Figure 18A) [169]. Alternatively, the primary rim can be selectively functionalized starting from the corresponding mercaptocyclodextrin using a radical thiol–ene reaction (Figure 18B) [170]. As an example of one of these polymerizations, we summarize in Figure 18C the synthesis of a 21-arm star polymer via a core-based approach, with the core being a molecule of β-cyclodextrin. To this end, heptakis [2,3,6-tri-O-(2-bromo-2-methylpropionyl]-β-cyclodextrin, prepared via the peracylation method described above, was treated with methyl methacrylate in the presence of copper bromide and n-propyl-2-pyridylmethanimine as a bidentate ligand for copper complexation.

**Figure 19:**
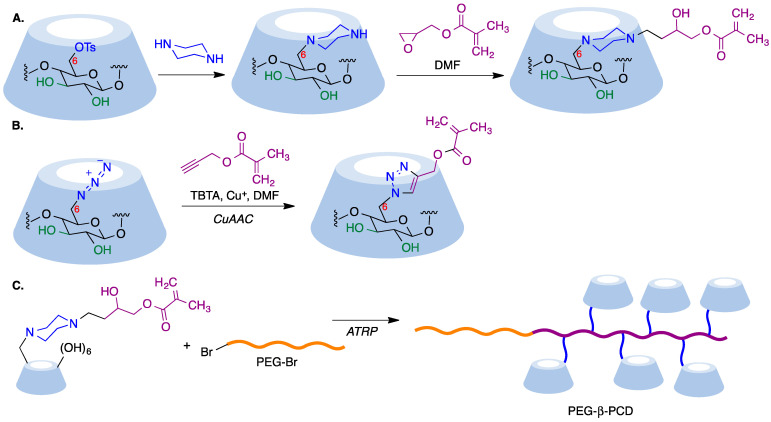
(**A**) Synthesis of a β-cyclodextrin derivative containing an acrylate moiety by opening of the epoxide moiety in glycidyl methacrylate with 6-monopiperazino-β-cyclodextrin. (**B**) Synthesis of a β-cyclodextrin derivative containing an acrylate moiety via a CuAAC reaction. (**C**) Synthesis of a CD-pendant polymer using the ATRP method.

In a reverse approach, the acrylate moiety required for the polymerization process can be introduced first into the cyclodextrin. Thus, treatment of 6-monopiperazino-β-cyclodextrin, obtained from the corresponding 6-monotosyl derivative, with glycidyl methacrylate afforded a mono-methacrylate substituted cyclodextrin via epoxide opening (Figure 19A) [171]. A similar type of starting material can be obtained by means of copper-mediated azide–alkyne couplings (CuAAC) of azido-cyclodextrins (Figure 19B) [172].

Mono-methacrylate cyclodextrins are useful as starting materials for radical polymerization reactions. Thus, poly(ethyleneglycol)-β-poly(cyclodextrin) (PEG-β-PCD), can be easily synthesized via atom transfer radical polymerization using a poly(ethylene glycol) macroinitiator (Figure 19C). This well-defined, hydrophilic, CD-pendant copolymer was able to include a variety of guest molecules and self-assembled into advanced nanostructures due to the synergistic effect of CD moieties [172].

**Figure 20:**
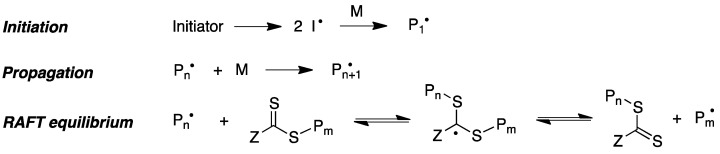
Simplified mechanism of the RAFT polymerization process.

Reversible addition–fragmentation chain transfer (RAFT) polymerization is another relevant modality of radical-induced polymerization that has proved to be an excellent tool to access a range of well-defined macromolecular architectures. This technique involves the use of a chain-transfer agent, usually a thiocarbonylthio compound, which helps to achieve control over the generated molecular weight and polydispersity during free-radical polymerization (Figure 20) [173].

**Figure 21:**
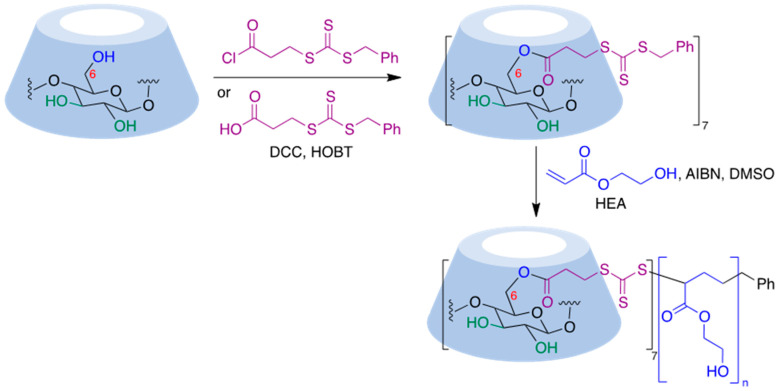
Application of the RAFT polymerization process to a cyclodextrin substrate.

Thiocarbonylthio functional groups can be installed onto cyclodextrin molecules by a simple acylation process using benzyl (3-chloro-3-oxopropyl) carbonotrithioate, an acid chloride [174], or the corresponding acid in the presence of dicyclohexylcarbodiimide (DCC) and hydroxybenzotriazole (HOBT) as a coupling reagent [175]. RAFT polymerization of this starting material in the presence of 2-hydroxyethyl acrylate (HEA), using 2,2′-azobisiobutyronitrile (AIBN) as initiator, afforded CD-based polymers with seven-arm star architectures [175] (Figure 21).

#### 9.5.2. Ring-Opening Polymerization (ROP)

**Figure 22:**
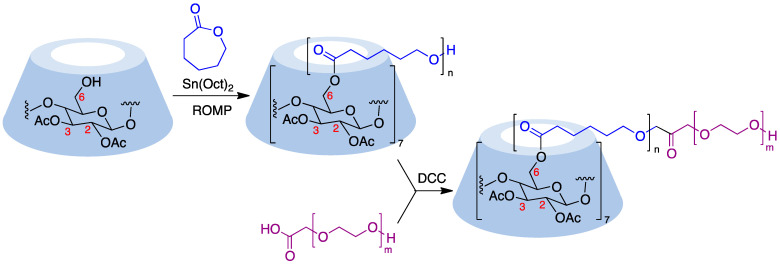
Ring-opening polymerization carried out on a cyclodextrin substrate.

Cyclodextrins can serve as core structures for anionic ring-opening polymerization (ROP) processes, which involve CD derivatization using cyclic esters [176]. Thus, treatment of β-cyclodextrin acetylated at its secondary rim with ε-caprolactone in the presence of Sn(Oct)_2_ as a catalyst resulted in polymers with narrow dispersity by esterification of the primary rim. The polymer arms were then coupled with DCC to carboxyl-terminated poly(ethylene glycol) to form amphiphilic copolymers that were able to undergo micellization (Figure 22) [177]. The ring opening polymerization of ε-caprolactone can also be carried out in the presence of wet β-cyclodextrin and has been performed in pressurized (12–13 MPa) batch reactors [178]. Lactide (3,6-dimethyl-1,4-dioxane-2,5-dione) is also widely employed to generate cyclodextrin-based polymers and, in the presence of 4-dimethylaminopyridine as an organocatalyst, has allowed the quantitative functionalization of α-, β-, γ-, and 2,3-dimethyl-β-cyclodextrins, with narrow molecular weight distributions (dispersity < 1.1) [179].

#### 9.5.3. CuAAC-Based Polymerization

**Figure 23:**
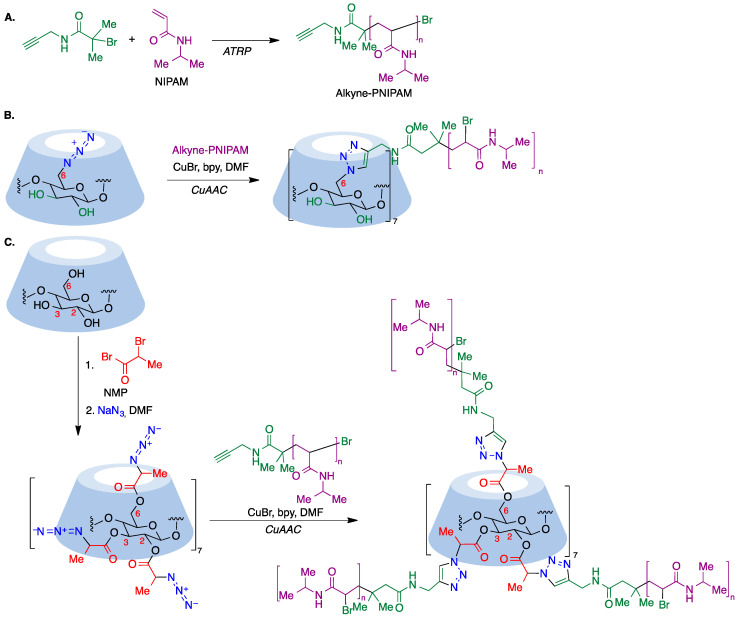
(**A**) Synthesis of PNIPAM, a polymer containing an alkyne moiety joined to poly(N-isopropylacrylamide. (**B**) Coupling of PNIPAM to a primary rim azido-CD via the CuAAC reaction. (**C**) Synthesis of a 21-star polymer via a CuAAC reaction of a per-azidocyclodextrin with PNIPAM.

Although ATRP is a suitable tool for the core-first synthesis of multiarmed polymers, it has some limitations because the polymerization of many arms from one core can lead to steric hindrance and a high local concentration of free radicals that facilitates star–star coupling and other termination events. This can be improved by use of the so-called grafting-to approach, where a functional core is coupled to a functional polymer, where the most extended reaction for such coupling is the copper-catalyzed azide–alkyne cycloaddition (CuAAC) [180], which is frequently associated with ATRP polymerization. To this end, a polymer containing an alkyne moiety joined to poly(N-isopropylacrylamide) (PNIPAM) is prepared via the ATRP polymerization technique (Figure 23A) and this precursor is then coupled to an azido-CD, which can be prepared via nucleophilic displacement of halogens at the primary rim of cyclodextrins (Figure 23B). In order to involve all cyclodextrin hydroxy groups, the primary rim can be modified via the CuAAC reaction and the secondary rim is used as the substrate of an ATRP or ROP polymerization. Alternatively, per-acylation with 2-bromopropionic bromide followed by bromide nucleophilic displacement with sodium azide affords a per-azidocyclodextrin, which is then submitted to a CuAAC reaction with the alkyne-PNIPAM polymer to furnish a 21-star polymer (Figure 23C) [181].

### 9.6. Cyclodextrin-Embedded Covalently Crosslinked Networks

**Figure 24:**
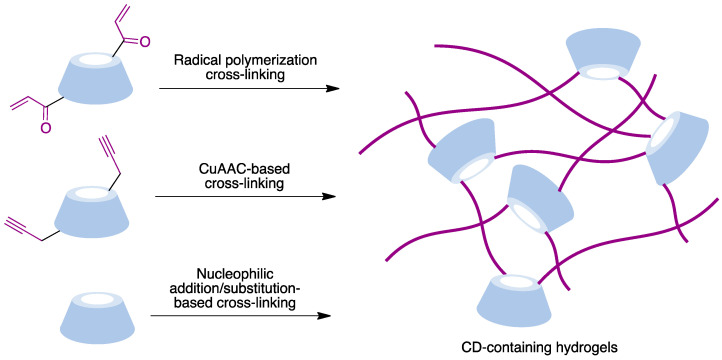
Main methodologies to access covalently CD-embedded hydrogels.

Hydrogels are three-dimensional crosslinked polymeric networks [182,183]. These biomaterials show unique characteristics such as their biocompatibility, water retention ability, and morphological similarity to bodily tissues that make them very useful for many biomedical applications. The incorporation of cyclodextrins, combining hydrophilic exteriors with hydrophobic inner pockets able to form inclusion complexes, provides an interesting approach to the design of hydrogels. The main crosslinking methods employed to prepare CD-based materials are summarized in Figure 24 and include radical polymerization, CuAAC-based polymerization, and polymerization processes based on nucleophilic addition or substitution reactions.

One interesting example of cyclodextrin-based networks are nanosponges, which can be described as colloidal structures that contain solid, three-dimensional, hyper-reticulated nanoporous structures that protect the entrapped molecules from degradation and help to improve the solubility of lipophilic compounds [184]. Cyclodextrin-based nanosponges (CDNSs), in particular, can be efficiently engineered for drug delivery purposes, especially in cancer therapy [185,186,187]. Indeed, due to the existence of lipophilic cavities in the cyclodextrin building blocks and the possibility to install a hydrophilic network in the nanosponge itself, depending on the nature of the crosslinker, these materials are ideal candidates to increase the stability of sensitive and volatile compounds, as well as the solubility of both lipophilic and lipophobic compounds [188,189].

**Figure 25:**
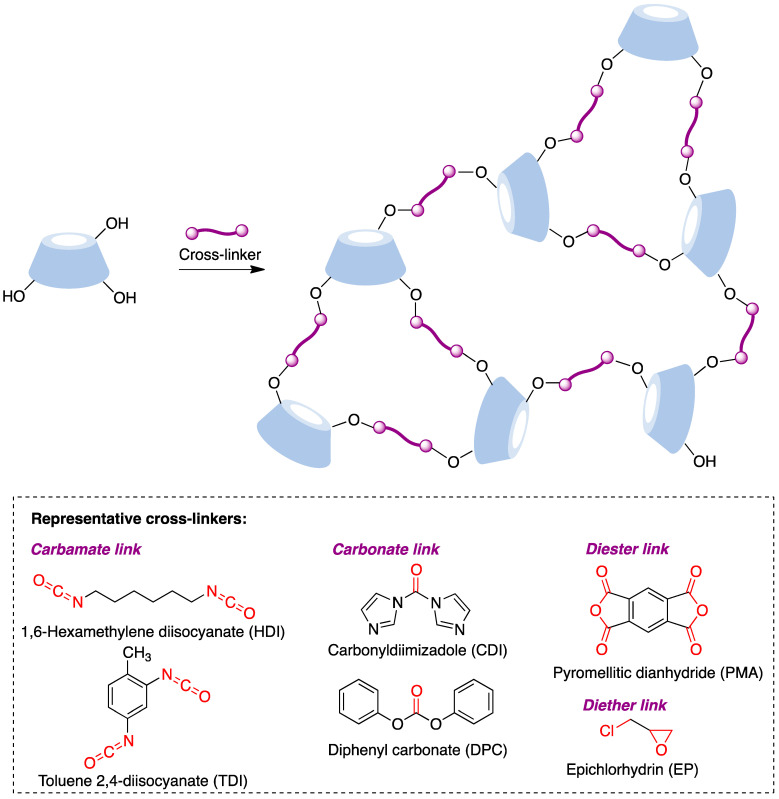
Structure and synthesis of CD-based nanosponges.

The synthesis of these nanostructures is achieved following the general scheme shown in Figure 25, where a suitable difunctional cross-linking reagent generates covalent bridges between cyclodextrin molecules by reacting with their hydroxyl groups to generate carbamate, carbonate, diester, or diether links. These transformations can be promoted under a number of experimental conditions, including, among others, solution conditions in conventional or non-conventional solvents, the joint melting of the CD and the linker [190], and ultrasound-assisted [191], microwave-assisted [192], and mechanochemical [193] conditions.

## 10. Analytical Aspects

### 10.1. Introduction

The characterization of inclusion complexes is usually carried out by combining several analytical techniques [194] that allow differentiating the included drugs from their free forms. For this purpose, it is important to study the possible changes observed in the experimental data obtained with the presumed inclusion complexes with respect to those obtained with pure guests, CDs, whose characterization has been reviewed by Szente et al. [195], or guest-CD physical mixtures. These changes in the measured instrumental magnitudes are either the result of alterations in the physical properties of the included compound or CDs, or result from the occurrence of chemical interactions (mainly hydrogen bonds, van der Waal forces, dipole–dipole electrostatic bonds, and hydrophobic interactions) between the guest and the host entities. Therefore, it is essential to apply sensitive and selective analytical techniques. Generally, techniques such as nuclear magnetic resonance or those performed on the complexes in the solid state present a lower sensitivity than that shown by techniques such as mass spectrometry or fluorimetry.

For studying the inclusion complexes, it is necessary to take into account the state in which they are obtained (solution or in solid state), with the procedure used for their preparation being critical [196], which strongly influences the properties of the presumed inclusion complex. Thus, Logadekar et al. [197] achieved the optimal inclusion complex of niclosamide-HP-β-CD by freeze drying compared with kneading or solvent evaporation procedures, while Araujo-Filho et al. [198] and Heimfarth et al. [199] found that the slurry complex method was the best one for the complexation of D-limonene with β-CD and of myrtenol with β-CD, respectively. Moreover, Shende et al. [200] obtained better results with respect to increased solubility and wettability of meloxicam when forming the inclusion complex with β-CD-based nanosponges instead of β-CD.

### 10.2. Analytical Techniques for Studying Inclusion Complexes in the Solid State

The solid CD inclusion complexes can be prepared [201,202] by removing the solvent from a solution of guest and CDs through co-evaporation, co-lyophilization, or spray drying. They can also be obtained by kneading a slurry of a physical mixture of the compound and CDs in the presence of a volatile solvent or a small volume of water that later is evaporated, or alternatively co-grinding the drug and CDs under microwave irradiation, sealed-heating using supercritical fluid, ultrasound, magnetic stirring, or high energy ball milling.

The main analytical techniques that can be used for the characterization of solid-state inclusion complexes include thermal analytical techniques, X-ray diffraction, electron microscopy, and spectroscopic methods [203]. Among them, the most commonly used are Fourier-transform infrared spectroscopy (FTIR), differential scanning calorimetry (DSC), scanning electron microscopy (SEM), and powder X-ray diffraction (PXRD) [202,203,204]. Nuclear magnetic resonance spectrometry (NMR) may be employed both for studying solid state inclusion complexes and isolated solid inclusion complexes and then solved in adequate conditions for NMR determination.

#### 10.2.1. X-ray Diffraction

This technique is suitable for characterization and identification of solid compounds, either crystalline or powder solids. It is a non-destructive technique that allows the sample to be used in further analyses via other analytical techniques. If the guest-CD inclusion complex crystallizes in stable single crystals of dimensions between c.a. 80 and 250 μm, these complexes can be studied via single-crystal X-ray diffraction (SCXRD), as in the case of the resveratrol/trimethyl-α-CD inclusion complex [205].

Inclusion complexes with these characteristics are difficult to obtain, and hence powder X-ray diffraction (PXRD) is used in most cases. PXRD does not require any sample pre-treatment since it is performed on a finely ground and homogenized sample and so the sample does not undergo any physical or chemical change during analysis. The diffractograms for the free guest, the single CD, the physical mixture, and the presumed inclusion complex are recorded and compared with each other. New peaks or variations in the intensity of the characteristic peaks of the pure compound may appear in the diffractogram of the presumed inclusion complex, showing the formation of a new crystalline species [202], as can be observed in the case of the (-)-borneol/β-CD inclusion complex [206], or a decrease in the crystallinity of the guest, such as in the case of the nifedipine/β-CD inclusion complex [86], which means a change in the properties of the drug due to the occurrence of interactions between the drug and the CDs. In other cases, in the diffractogram of the presumed inclusion complex, the loss of the crystalline structure of the pure compound and its transformation into an amorphous structure are observed, while the diffractogram of the physical mixture usually retains the representative peaks of the single CD and the free drug, although with less intensity. These observations can be interpreted as evidence of the formation of a true inclusion complex as observed for the niclosamide/HP-β-CD inclusion complex [198], meloxicam/β-CD-based nanosponges [201], and genistein/amino acid appended β-CD [79]. These results were corroborated by the experimental data obtained using other analytical techniques, especially DSC.

#### 10.2.2. Thermal Analytical Techniques

These are a set of techniques that, by subjecting the sample to controlled heating, allow the study of processes that involve a change in enthalpy such as crystallization, melting, or vaporization, or a change in weight like dehydration and volatilization, as well as their thermal stability (degradation or decomposition). Differential scanning calorimetry (DSC) and thermogravimetric analysis (TGA) [207] provide relevant information for the characterization of drug-CD inclusion complexes. For this purpose, it would be ideal for the guest drug to be a crystalline solid with a melting or boiling point below the thermal degradation range of CD or to be volatile between 60 and 250 °C to allow the evaluation of the formation of a real inclusion complex with CDs. Thermograms of the CDs show two clearly differentiated zones, the first one between 90–130 °C corresponding to the loss of water molecules housed in the CD cavity, and the second one at temperatures above 300 °C, corresponding to their thermal decomposition [202].

In the cases of compounds whose melting points are in the dehydration range of the CDs, these events overlap in the DSC thermograms and cannot be distinguished. A complementary technique such as hot-stage microscopy (HSM) can be of assistance, since with this technique it is possible to observe the appearance of bubbles corresponding to the evaporation of water within the molten solid. This technique also allows to observe phase changes/recrystallizations, which are hardly visible in DSC thermograms. HSM was employed in conjunction with DSC for the study of the resveratrol/trimethyl-α-CD inclusion complex [206]. It is possible to evaluate the formation of inclusion complexes between CDs and amorphous solids if there is an increase in the decomposition temperature in the thermogram of the inclusion complex with respect to that observed in the thermogram of the pure drug or the physical mixture because complex formation provides an increased thermal stability to the included drug molecule [202].

Differential scanning calorimetry (DSC) is the most widely used of all thermal methods and one of the most powerful analytical tools employed in the solid-state characterization of compounds. DSC is a calorimetric method in which differences in energy (heat flow) are measured under a controlled-temperature program. The thermogram of the free drug, if it is a crystalline solid, will show a well-defined sharp endothermic peak corresponding to its melting point, and if it is a volatile compound it will show a peak representing its volatilization. In the case that it also presents polymorphism, exothermic peaks corresponding to recrystallization processes would be observed. Finally, a descending zone will appear showing the thermal decomposition stage. All these events allow the accurate characterization of the drug. The thermogram of the physical mixture presents peaks due to both the drug and the CD clearly showing the peak corresponding to the melting point of the drug, as well as the dehydration stage of the CD. The complete loss of the melting point peak of the drug in the thermogram of the presumed inclusion complex would be evidence for the formation of a true inclusion complex [198,201]. Other changes that can be observed in this thermogram are a shift to lower temperatures and/or lower intensity in the dehydration zone of the CD and a shift to higher temperatures of the thermal decomposition of the included drug [87]. Thus, in the case of the usnic acid/β-CD inclusion complex, the disappearance of the melting point peak is accompanied by a decrease in the intensity of the β-CD dehydration band and an increase in the thermal stability of the usnic acid [208].

In the DSC study of 17-α-methyltestosterone and β-CD inclusion complexes, it was observed that when the inclusion complex was prepared by lyophilization the peak corresponding to the melting point of the free drug disappeared completely, whereas when prepared by kneading, the peak was observed but with a much-decreased intensity with respect to the free hormone. In addition, the dehydration zone of the β-CD shifted to lower temperatures along with a decrease in its intensity, indicating the loss of water molecules upon inclusion of 17-α-methyltestosterone into the CD cavity [83]. The same behavior was observed for the binary and ternary inclusion complexes of dapsone with HP-β-CD (melting point peak of the free drug is missing) and β-CD (melting point peak decreases in its intensity) [209].

In thermogravimetric analysis (TGA), the loss of mass of the sample during a controlled heating program under a controlled atmosphere is recorded, arising as a consequence of processes such as dehydration, volatilization, or degradation. It is a useful technique for monitoring the process of inclusion of a drug into the CD cavity since it is possible to observe whether the dehydration stage of the CD or the volatilization of a volatile drug is modified, or if the thermal stability of both the CD and the drug is modified. Thus, Dalmolin et al. [210] studied the thermal stability of β-CD and modified β-CD with hexanoic anhydride using this technique, finding that the latter increases its stability since the degradation temperature is shifted from 325 °C in β-CD to 354 °C in chemically modified β-CD. Moreover, in the case of the modified CD, dehydration is not observed, indicating that this CD presents higher hydrophobicity. Trindade et al. [211] formed inclusion complexes between carvacrol (a volatile monoterpene) and β-CD via two different procedures: freeze drying and ultrasound. The formation of these inclusion complexes was evidenced using several analytical techniques, including thermal analytical techniques, such as DSC, TGA, and DTG (differential thermogravimetry). In the thermograms of the inclusion complexes obtained by both procedures, the disappearance of the broad peak corresponding to the volatilization of carvacrol (32–169 °C) was observed. Cerutti et al. complemented the studies of the nifedipine/β-CD inclusion complex with and without aspartic acid using DSC and TGA, with the data collected being in agreement with those obtained via PXRD [86]. It was observed that aspartic acid contributed to the formation/stabilization of the inclusion complex since nifedipine decreased its crystallinity by around 50% in multicomponent systems.

#### 10.2.3. Electron Microscopy

Morphology and surface characterization of the solid particles of the inclusion complexes can be mainly carried out by means of scanning tunneling microscopy (STM) and scanning electron microscopy (SEM); the latter being the most commonly used for this purpose. Atomic force microscopy (AFM) provides extensive surface information that other electron microscopy techniques cannot reach.

Although scanning electron microscopy (SEM) is not suitable to confirm the formation of a true inclusion complex, it nevertheless provides morphologic and topographic information about the surface of a solid, complementing the information obtained via X-ray or thermal analysis. The samples must be coated with gold or another metal element under vacuum prior to analysis and this fact could alter the solid, therefore being a disadvantage of this technique. The use of SEM enables the determination of the size, shape, and homogeneity of the particles of the solids studied, helping to find the most optimal procedure to obtain the inclusion complex [86,201] or revealing the micro-morphologic changes suffered by the guest and the host when the inclusion complex is formed. Dapsone changed from irregular orthorhombic crystals to well-defined spherical particles when it interacted with HP-β-CD [210]. Genistein appeared as large cuboidal particles with claviform crystals of different size and amino-appended β-CD as hollow spherical particles; however, their inclusion complex appeared as an irregular massive aggregate, which was very different from the physical mixture [79]. Scanning probe microscopy, AFM and STM, make it possible to visualize surface details of solids at the atomic level, achieving an average resolution of 20 Å [212].

Tapping-mode AFM is adequate for distinguishing molecules with similar morphological features but different local mechanical properties, such as CD inclusion complexes and their hosts. Thus, we observed [213] that, as a consequence of complexation, retinal/β-CD nanotubes showed a higher local rigidity than those coming from β-CD. Desai and Prabhakar studied the surface topography of cilostazol/HP-β-CD inclusion complexes, observing a significant change in the morphology of the surface: from rough in the case of the free cilostazol to smooth in the case of the inclusion complex due to the guest–host interactions [214].

#### 10.2.4. Spectroscopic Techniques

Spectroscopic techniques, such as Fourier-transform infrared spectroscopy (FT-IR), attenuated total reflectance (ATR)-FT-IR spectroscopy and Raman spectroscopy, are valuable analytical techniques to evidence and characterize solid state compounds.

Fourier-transform infrared spectroscopy (FT-IR) is largely employed for qualitative organic analysis and structure determination due to its high degree of structural information and intermolecular hydrogen bonding association. Each organic compound presents characteristic absorption bands in the IR spectrum. Therefore, changes in the spectrum of the drug such as an increase or decrease in the intensity of the bands, broadening or shifts in the bands, or the disappearance of some bands may be indicative of the formation of the inclusion complex, suggesting which bonds or functional groups of the drug may be involved in the interaction with the CD cavities. Despite the fact that the main characteristic bands of the free drug are often overlapped or masked by the bands of the CDs, there are certain bands corresponding to carbonyls, carboxyl, nitrile, and amine groups that can be observed and shifted when the inclusion occurs; these shifts and shape changes are not observed in the corresponding physical mixture [202,205]. Thus, the FT-IR spectrum corresponding to meloxicam/β-CD-based nanosponges inclusion complex showed shifts in the aromatic peaks together with an intense broad peak at 3429.7 cm^−1^ that indicate that the aromatic ring of meloxicam penetrates into the hydrophobic cavity of the β-CD, and then forms hydrogen bonds with the nanosponges [201]. In the case of sulfamethoxazole/HP-β-CD, the absence of SO_2_ stretching bands is indicative of the interaction of this moiety with the CD cavity [215].

Attenuated total reflectance Fourier-transform infrared spectroscopy (ATR-FT-IR) combines the selectivity and sensitivity provided via FT-IR with the reproducibility, simplicity, and speed of ATR, in addition to being a non-destructive technique. The spectra show the attenuation of the evanescent wave produced by the absorption of this REM by the sample, which is independent of the sample thickness. These spectra are similar to those obtained via FT-IR, but the relative intensity among the peaks or bands are different. The sample handing is very easy, just it is placed and pressed on opposite sides of a transparent crystalline material of high refractive index, thus avoiding the disadvantages derived from the preparation of KBr tablets for conventional FT-IR measurements.

ATR-FT-IR is very useful for studying intra- and inter-molecular hydrogen bonds, allowing to probe the formation of the inclusion complex and showing the specific functional groups implicated in the guest–host interaction [202]. Thus, comparing the spectral changes observed in the O-H stretching band between the physical mixture and the idebenone/HP-β-CD inclusion complex, Venuti et al. [216] demonstrated the involvement of OH groups of idebenone in new guest–host interactions through the formation of a drug-CD hydrogen bond. The complexation was also proven by the relevant changes in the C=O and C=C stretching bands associated with the breakdown of intermolecular hydrogen bonds of the idebenone. ATR-FT-IR has been employed to study different CD-based nanosponges loaded with different drugs, such as repaglinide or dexamethasone [213].

Raman spectroscopy is a complementary technique to FT-IR that measures the inelastically scattered radiation by the molecules of the compound under study. Raman spectra also have functional group and fingerprint regions that permit the identification of specific compounds. In these spectra, the appearance of a peak usually corresponds to a mode of vibration accompanied by a change in the polarizability of the molecule. This technique is useful to study drug-CD inclusion complexes because the inclusion of the guest molecule in the CD cavity leads to changes in the polarizability together with changes in the ring size or/and a decrease in the vibrational relaxation time of the included functional groups, which results in changes in the Raman peaks of the drug and the CD.

As in FT-IR, the peaks from CD Raman spectra overlap the guest Raman peaks. However, there is a zone between 1500–1750 cm^−1^, corresponding to stretching vibrations of C=O and C=C of the aromatic rings, where CDs present no peaks, which enables observation of the changes in the intensity and mainly the shifts in the wavenumber produced in the characteristic peaks of the drug molecules after inclusion. This region of the Raman spectrum was used to evidence the formation of inclusion complexes of sulfamethoxazole with HP-β-CD and β-CD [216] and the inclusion complex of idebenone with HP-β-CD [217].

### 10.3. Analytical Techniques for Studying Inclusion Complexes in Solution

In the case of the inclusion complexes in solution, there is a dynamic equilibrium between the free drug and the host. This makes it possible to carry out tests of entrapment efficiency, solubility, stability, or monitoring of reactions that may be altered by the interaction of the drug with the CDs, such as the photo-isomerization of retinoids [13], the hydrolysis of the camptothecin lactone ring [50], and acid–base equilibrium in norharmane [217].

The main analytical techniques that can be used for the characterization of inclusion complexes in solution include spectroscopic, electroanalytical, and separation techniques. Among them, the most commonly used being ultraviolet/visible (UV-Vis) absorption spectroscopy, fluorescence spectroscopy, high-performance liquid chromatography (HPLC) and, above all, nuclear magnetic resonance spectrometry (NMR). Other techniques can also be used for this purpose, such as mass spectrometry, circular dichroism, polarimetry, isothermal titration calorimetry, or dynamic light scattering [204,218]. Molecular modeling techniques help spectroscopic techniques in the elucidation of the three-dimensional structure of the complex, establishing the points of interaction between the drug and the CD, as well as the stability of the inclusion complex. The experimental characterization of the complex stability is concluded by determining its association constant and stoichiometry. Kfoury et al. summarized the main analytical tools employed for the determination of association constants and stoichiometry for CD inclusion complexes [2,219]. It is important to note that the analytical method chosen for the calculation of the drug/CD association constants, as well as the concentration range used in the assay, influences the value found [220].

#### 10.3.1. UV-Vis Absorption Spectroscopy

This technique is widely used for the calculation of association constants and stoichiometry of inclusion complexes, as well as for solubility assays or dissolution tests, drug release studies, and encapsulation efficiency. Although UV-Vis absorption spectrophotometry has limited application for qualitative analysis, it is useful for detecting the presence of certain functional groups that act as chromophores. Therefore, it helps to evidence the formation of the inclusion complex—whether the chromophore group of the drug molecule interacts or is included in the CD cavity—passing into an environment of higher hydrophobicity and thus producing an effect similar to the solvatochromic effect produced by the change in the polarity of the solvents.

Upon interaction of the drug with the CDs, changes are observed in the absorption spectrum of the free drug, which can be in the intensity of the peaks, shifts of the absorption maxima, or appearance of new peaks that were not usually observed under the experimental conditions used. Thus, we studied the formation of inclusion complexes of β-carboline alkaloids harmane and harmine with β-CD and chemically modified β-CDs via UV-Vis absorption spectrophotometry in conjunction with spectrofluorimetry and NMR [14]. The β-carboline ring possess an acidic nitrogen, which loses its proton in alkaline media. In the UV-Vis absorption spectra of harmane and harmine in aqueous solution (pH 7.2–7.9, close to their p*K*_a_ values), only the band corresponding to the cationic form is observed as a consequence of the rapid proton transfer with the solvent. However, in their UV-Vis absorption spectra in ethanol, on the contrary, only the band corresponding to the neutral form is observed, as it corresponds with a lower polarity environment than water. UV-Vis absorption spectra of the complexes evidence that the inclusion of these alkaloids in the CD cavities hampers the proton transfer and decreases the formation of the cationic form of these alkaloids. The environment provided by CD is partly reminiscent of that provided by ethanol. In the case of the niclosamide inclusion complex with HP-β-CD, only a hyperchromic effect is observed at its absorption maximum with respect to the UV-Vis absorption spectrum of the free drug [198].

#### 10.3.2. Fluorescence Spectroscopy

This technique is characterized by its good selectivity and sensitivity, being a potent tool to verify the efficiency of the formation of the drug/CD inclusion complex. It is also employed for the calculation of association constants and stoichiometry of inclusion complexes, as in the case of the inclusion complexes of camptothecin and luotonin A with β-CD and HP-β-CD [50] and the inclusion complexes of harmane and harmine with β-CD and chemically modified β-CDs [14].

Generally, it is observed that the formation of the inclusion complexes produces an increase in the fluorescence emission intensity and/or in the fluorescence quantum yield of the drug due to the fact that the radiationless deactivation processes are notably decreased, resulting in enhancement of the fluorescence emission. It may also be accompanied by shifts in the fluorescence emission maxima or the appearance of new peaks that were not usually observed for the free drug under the experimental conditions employed.

The formation of inclusion complexes of the antitumor alkaloids camptothecin and luotonin A with β-CD and HP-β-CD was verified spectrofluorimetrically via titration of these alkaloids with increasing amounts of both CDs in aqueous solution at strongly acidic pH values [50]. The fluorescence emission spectra of both alkaloids in aqueous solution at pH 1 show the characteristic maxima of the cationic forms and a shoulder corresponding to the neutral forms, while the corresponding spectra of the inclusion complexes with the studied CDs show, in addition to the maxima of the cationic forms whose intensity hardly varies, the appearance of the maxima of the neutral forms whose intensity increases as the amount of added CD increases. Thus, the more lipophilic neutral form is included in the CD cavity, allowing it to be observed in strongly acidic media.

Furthermore, inclusion complexes of sulfamethoxazole with β-CD and HP-β-CD were also studied via fluorescence spectroscopy [216]. This drug presents an aniline moiety and an isoxazole ring localized at the end of its structure, which can act as an electron donor-acceptor system. The fluorescence emission spectrum of free sulfamethoxazole in aqueous solution shows only one intense peak, centered around 350 nm. The addition of increasing amounts of HP-β-CD to an aqueous solution of sulfamethoxazole leads to the appearance of an additional broad band (centered at 425 nm) in the fluorescence emission spectrum due to the intramolecular charge transfer process that is favored by the inclusion of the drug inside the cavity, in addition to the main emission band of the sulfamethoxazole, the intensity of which increases with the amount of added CD.

#### 10.3.3. Nuclear Magnetic Resonance Spectrometry (NMR)

NMR is one of the most valuable analytical tools for the characterization of inclusion complexes, as it provides insight into the interactions that occur between the drug and the CD, the structure of the inclusion complex, and the orientation of the molecule within the cavity of the CD. This is due to the fact that the proximity between groups and the interactions among the hydroxyl groups of CDs and the drug molecules cause changes in the chemical shifts as well as in the shape of the signals. These changes are more pronounced in the parts of the molecule that are inside the CD cavity.

NMR spectrometric techniques suitable for the study of CD complexes include, among others, the well-known ^1^H NMR and ^13^C NMR, diffusion-ordered spectroscopy (DOSY), nuclear Overhauser effect spectroscopy (NOESY), and rotational Overhauser effect spectroscopy (ROESY).

^1^H NMR and ^13^C NMR are very powerful techniques to study the interaction between drug molecules and cyclodextrins because the chemical and electronic environments of the protons or the carbons, respectively, are affected by CD complexation, which causes changes in the chemical shifts registered in the inclusion complex spectrum with respect to the free drug and cyclodextrin spectra.

DOSY involves the measurement of diffusion coefficients and it is a technique suitable to study inclusion complexes and show binding between molecules of very different sizes. When a drug is included inside the host cavity, the diffusion coefficients for both drug and host decrease. Nuclear Overhauser effect-based techniques are very useful to explore the existence of intra- and inter-molecular interactions, if the atoms are in spatial vicinity (3–5 Å), helping to clarify the three-dimensional structure of the inclusion complex and providing information about inter-molecular distances [219]. Due to the molecular weight of cyclodextrins, ROESY experiments are usually more sensitive than NOESY.

By simple comparison of the ^1^H NMR spectra of CDs and the presumed inclusion complex, changes in the chemical shifts of the H-3 and H-5 protons of the CDs (located inside the cavity) prove that the drug molecule has been included inside the CD cavity, as it occurs in the inclusion complex of methyltestosterone and β-CD [83]. If the change in chemical shift occurs only at the H-3 proton, it means that the cavity inclusion is shallow. This was observed in the case of the inclusion complex of cilostazol with HP-β-CD [215]. In addition, in this case, the differences observed between ^1^H NMR spectra of free cilostazol and the inclusion complex revealed the partial inclusion of the cyclohexane ring and tetrazole moiety of this drug in the HP-β-CD cavity, which was also corroborated via 2D-ROESY and molecular modeling experiments.

For the study of inclusion complexes of camptothecin [50] with β-CD and HP-β-CD, in addition to ^1^H RMN, ^13^C RMN was used. Comparing the ^13^C RMN spectra for the free camptothecin and its inclusion complexes with these CDs, the more intense changes were observed in the signals corresponding to the carbons of the quinoline ring of the camptothecin, which is the most lipophilic part of the molecule, supporting that the quinoline moiety is placed inside the CD cavity. ^1^H RMN, docking, and molecular dynamics computational studies confirm the full insertion of this part of the molecule in the CD.

β-Carboline alkaloids norharmane, harmane, and harmine inclusion complexes with β-CD and HP-β-CD were characterized using ^1^H NMR, ^13^C NMR, and 2D ROESY [15]. From the study of the ^1^H NMR spectra of the inclusion complexes, it can be observed that the signal corresponding to the water molecules included in the CD cavity disappears and that the signals due to the protons of the -OH at the rims of the CDs are shifted, indicating that the inclusion of these compounds takes place in the CD cavity. The ^13^C NMR spectra study allowed to distinguish the formation of 1:1 and 1:2 β-carboline/CD inclusion complexes and to propose that the benzene rings of these drugs enter the CD cavity first since they are the most lipophilic part of these molecules. A study of the 2D-ROESY spectra revealed that, in the case of norharmane, the benzene and pyridine rings can be included in the CD cavity with similar probability since it lacks the methyl group on the pyridine ring as harmane and harmine.

Venuti et al. [217] employed ^1^H NMR, 2D-ROESY, and DOSY to characterize the idebenone/HP-β-CD inclusion complex. The 2D-ROESY assay revealed that the inclusion occurs through the quinone ring of the molecule. DOSY experiments pointed out that the diffusion coefficients for the drug and the HP-β-CD decreased when the inclusion complex was formed, thereby supporting the results obtained via both ^1^H NMR and 2D-ROESY.

NMR can also be employed for the calculation of association constants and stoichiometry of inclusion complexes. In the case of the association constants, however, discrepancies can be found in the values obtained via NMR with those obtained using other techniques due to the concentration range or the solvent employed for obtaining the NMR spectra. Thus, in the methyltestosterone/β-CD inclusion complex [83], the association constant was calculated using DOSY (in D_2_O/DMSO) and solubility isotherm (in water via UV-Vis spectrophotometry), obtaining values of 2846 L mol^−1^ and 8045 L mol^−1^, respectively.

#### 10.3.4. Separation Techniques

Gas chromatography (GC), capillary electrophoresis (CE), and, mainly, high performance liquid chromatography (HPLC) are widely used for the calculation of association constants and stoichiometry of inclusion complexes, as well as for solubility assays or dissolution tests, encapsulation efficiency, and stability studies [195,197,219,220]. HPLC with evaporative light scattering detection is useful to evaluate the formation of CD aggregates [221] and to quantify CDs as excipients in pharmaceutical formulations [222].

In the case of volatile compounds/CD inclusion complexes, GC can be employed for their quantification and calculation of their association constants using static headspace gas chromatography (SH-GC), in which the volatile compound is in equilibrium between the gas phase and the condensed liquid (or solid) phase in a closed vial [2]. SH-GC achieves improved sensitivity and LODs in the determination of these compounds that exhibit low solubility and stability. Lima et al. [203] reviewed the preparation, characterization, and pharmacological characteristics of terpenes/CD inclusion complexes.

HPLC is the most widespread technique for analytical separations in many different fields, including pharmaceutical and food industries, due to its sensitivity, its accuracy quantitative determination of both volatile and non-volatile compounds, its versatility, and its ability to separate members of a homologous series and, under certain experimental conditions, isomers of chiral substances.

Shende et al. [201] carried out the study of solubility and encapsulation efficiency of meloxicam inclusion complexes with β-CD and β-CD-based nanosponges via HPLC-UV-Vis detection, using a C18 column as the stationary phase and a mobile phase composed of acetonitrile and phosphate buffer 60:40 *v*/*v* (pH = 3.4). From the results obtained, it can be deduced that encapsulation with the nanosponges produces a significant improvement in the solubility and dissolution rate of meloxicam. Therefore, nanosponges are promising and interesting carriers for the controlled release of this drug to increase its pharmacology activity.

An HPLC-UV-Vis detection (C18 column as stationary phase; mobile phase composed of water/acetonitrile 25:75 *v*/*v*) method has been validated for the quantitation of idebenone and its inclusion complex with HP-β-CD, the study of solubility, and the determination of the inclusion complex association constant. The encapsulation of this drug inside the CD cavity achieves an important increase in its solubility in water, demonstrating that HPβ-CD could be a good delivery system for idebenone [217].

In the cases described above, the solutions analyzed were prepared from drug solutions at a fixed concentration to which different amounts of CD were added to prepare inclusion complex solutions with different molar ratios. Therefore, these analytical methods are more laborious and time-consuming.

There are other strategies to approach the study of drug/CD inclusion complexes via HPLC, which involve using the CDs in the stationary phase or adding them to the mobile phase acting as mobile phase additives (MPAs) [223,224]. When CDs are used as MPAs, an increase in the selectivity of the separation is achieved as a consequence of a secondary chemical equilibrium between the drug and the CDs associated with the chromatographic separation equilibrium. In addition, by improving the solubility of the analytes and modifying their retention properties, CDs as MPAs also improve efficiency and reduce retention factors. Another advantage of their use as MPAs is that they allow the use of a higher proportion of water in the mobile phases and the substitution of acetonitrile by less toxic organic solvents such as ethanol or methanol. This leads to a more sustainable and environmentally friendly HPLC method [225].

It is important to identify the association constants between the CDs and the analytes to be separated in order to evaluate the best experimental conditions to achieve good efficiency and resolution in the chromatographic separation. For the separation of three chemically and structurally related compounds (norharmane, harmane, and harmine) via RP-HPLC with fluorometric detection using TMβ-CD and DMβ-CD as MPAs, the association constants of the inclusion complexes were calculated. Two types of stationary phases (C1 and C18) and two hydroalcoholic mobile phases (ethanolic and methanolic) were also studied. With these MPAs, an organic solvent decrease of up to 50% was achieved with good resolution and efficiency [226]. The analysis of these beta-carbolines in human serum samples via RP-HPLC with fluorometric detection was developed and validated, employing a C18 column and native β-CD and HPβ-CD as MPAs [15]. Alternatively, employing a C1 column as the stationary phase and hydroalcoholic mobile phases (ethanolic and methanolic) with native β-CD and HPβ-CD as MPAs [227], a significant reduction in organic solvent (50–70%) was achieved.

Moreover, CDs are frequently employed as chiral selectors in capillary electrophoresis (CE) and their potential and analytical possibilities have been recently reviewed [228,229,230]. The enantioseparation is achieved by the partial or total inclusion of the chiral compound in the CD cavity and the establishment of secondary interactions with hydroxyl groups on the rim of the CD. Discrimination between enantiomers will occur if they exhibit different electrophoretic mobility as a result of their different inclusion complex association constants. A greater difference in electrophoretic mobility will contribute to higher resolution.

An important requirement for the CE separation is that either the analyte, the CD, or both must be charged and, thus, possess different electrophoretic mobility. Thus, a quantitative determination for lercanidipine enantiomers [231] via CE-UV-Vis detection in pharmaceutical formulations was developed and validated. Since this drug has a basic character, it was worked at pH = 4 so that it would present a positive charge and move toward the cathode. The best separation was obtained by adding uncharged CDs, specifically 10 mM TMβ-CD, to 200 mM of sodium acetate buffer as the background electrolyte. In this way, a green method was achieved by not using toxic organic solvents or pollutants, with good efficiency and resolution, and analysis times of less than 15 min. Szabó et al. [232] developed and validated a CE-UV-Vis-based method for the enantioseparation of asenapine maleate using 7 mM β-CD as the chiral selector and 160 mM of TRIS-acetate buffer as background carrier at pH 3.5 in order to maintain the drug in a charged state. However, in the case of enantiomeric separation of four benzodiazepines [233] via CE with UV-Vis detection, sulfated CDs (anionic CDs) were used as chiral selectors because the benzodiazepines are neutral in the pH range 2,9–11,6. The best resolution was achieved with 5% heptakis-6-sulfate-β-CD and methanol as the organic modifier added to borate buffer (pH = 9). In these conditions, a very high efficiency is obtained due to the stacking induced by the opposite direction of the electrophoretic mobility of the sulfated-CD to the electro-osmotic flow and the neutral drugs that move with the buffer. When the benzodiazepine/CD inclusion complex is formed, the electrophoretic mobility of the drug decreased, allowing better resolution and efficiency in the chiral separation. The benzodiazepine/CD inclusion complex association constants were also calculated, revealing differences between the enantiomers of the benzodiazepines studied and leading to different mobilities between the free drug and its inclusion complex.

#### 10.3.5. Other Techniques

Circular dichroism and polarimetry are two attractive analytical techniques that allow distinguishing and quantifying chiral substances due to their optical activity of deviating the plane of polarized light. In the case of circular dichroism, it is possible to take advantage of the chirality of CDs to also analyze non-chiral substances since when the inclusion complex is formed, it induces this property of optical activity (induced Cotton effect), with the peak being observed instead of in the characteristic zone of the CD (below 220 nm) but at the wavelength of the UV-Vis absorption maximum of the included compound. In addition, depending on the positive or negative sign of the circular dichroism signal, it is possible to determine whether the compound inclusion has occurred parallel (positive Cotton effect) or transverse (negative Cotton effect) to the longitudinal axis of the CD, providing complementary information on the configuration of the inclusion complex. Moreover, this technique enables the calculation of association constants and stoichiometry of inclusion complexes [219,234]. The Cilostazol/HPβ-CD inclusion complex was studied using circular dichroism, taking advantage of the Cotton effect induced by the CD, which revealed a positive band at 260 nm (drug absorption maximum) in the circular dichroism spectrum. The positive signal indicated that the inclusion of the compound was parallel to the longitudinal axis of the CD through its tetrazole moiety [215].

Mass Spectrometry (MS) is one of the most widespread employed analytical techniques because it allows structure determination leading to an exact identification of the studied compounds together with a very sensitive quantitation. It is frequently used in tandem with separation techniques, GC-MS, HPLC-MS, and CE-MS, thus increasing the performance of both techniques. One of their applications is the study of inclusion complexes to determine the stoichiometry of the complex and to carry out solubility tests since it is able to differentiate between the free drug and the included drug. In spite of there being many different modes/techniques for compound ionization, it is necessary to select the “soft” ionization modes in order to not alter the supramolecular entity drug-CD. For that reason, in the study of CD inclusion complexes, the most efficient soft ionization mode is electrospray ionization (ESI) due to the fact that the interactions between the drug and CD are non-covalent [204]. ESI-MS has been applied to study inclusion complexes with sulfamethoxazole [216] and β-CD or HPβ-CD, and HPβ-CD inclusion complexes with luotonin A and camptothecin [16], asenapine/β-CD [233], curcumin/β-CD, baicalein/β-CD, and ferulic acid/HPβ-CD [195].

Isothermal titration calorimetry is a thermal analytical technique that enables recording of the thermal changes produced by the drug/CD interaction when aliquots of CDs are added to the aqueous solution of the drug, providing in a single experiment the determination of entropy and enthalpy changes and the possibility of the determination of inclusion complex association constants and stoichiometry. Some applications of this technique for the study of CD inclusion complexes with drugs such as procaine hydrochloride, ibuprofen, valsartan, and flurbiprofen have been described [204,208].

Dynamic light scattering (DLS) is the technique of choice for particle size measurement. It is also frequently used for drug release studies and for the control of the manufacture of CD-based nanofibers. Doxorubicin/β-CD and hydrocortisone/γ-CD have been analyzed by this technique [204]. DLS allows to evidence the formation of CD aggregates and to calculate the critical aggregation concentration, which is important information in the preparation of pharmaceutical dosage forms [222].

Another group of attractive techniques for the characterization and quantification of CD inclusion complexes with electroactive drugs is electroanalytical techniques, among which the most widely applied in this field are polarography and voltammetry. These two techniques are affordable, sensitive, and selective and the latter can be coupled with HPLC. Moreover, in voltammetry, the amount of analyte required for the analysis is minimal and current measurements are made as a function of the applied potential under conditions that increase the polarization of the working microelectrode. Applications of voltammetry, polarography, potentiometry, and conductimetry for the study of drug/CD inclusion complexes and the determination of their association constants are discussed by Mura [219]. The drugs analyzed include nifedipine, lumazin, bupivacaine, or ibuprofen.

Table 3 summarizes the preparation methods as well as the analytical techniques used for the characterization of different drug/inclusion complexes, with emphasis on whether the complexes are in solid state or in aqueous solution. In addition, the technique used for the calculation of the association constants as well as for the stoichiometry determination is indicated.

### 10.4. Analytical Techniques for Determining Drug Loading and Encapsulation Efficiency

The formation of drug–cyclodextrin or drug–polymer complexes based on cyclodextrin requires different analytical methodologies that provide different but complementary information on the existence of these supramolecular aggregates. The multiple experimental methodologies for the formation of drug-CD complexes make it necessary to select the appropriate analytical techniques to characterize them. Thus, once the complexes have been obtained, they can be precipitated and isolated in the solid state or kept in solution, and the existence of these drug-CD complexes can be demonstrated by means of characterization techniques in solution.

While increasing the stability or solubility of drugs contributes to improving their bioavailability, it is important that this relevant property is enhanced by the physical nature and size of the particles that make up the active pharmaceutical ingredient encapsulated in CDs. Therefore, microscopy techniques are frequently used to determine the particle size, or aspects related to the granulometry and porosity measurement of the drug-CD aggregates. Different analytical techniques have been detailed above for characterization of complexes in solution

However, it is important to remark that an increase or improvement in bioavailability involves comparing the plasma concentrations of the dosage form in use with the drug-CD aggregates. This implies that for comparison purposes, the exact “load” of the drug that has been trapped by the CD or CD polymer must be known. This aspect must also be considered since the analytical technique employed for the quantification of the released drug as well as the extraction procedure are very important.

To determine the entrapment/encapsulation efficiency, the inclusion complex has to be dissociated using an organic solvent (usually methanol, acetonitrile, or isopropyl alcohol) and kept in sonication for a time that can vary from a few minutes to one or two hours; it is then centrifuged and the fraction of drug in the organic solvent is quantified. Quantification can be performed via UV-Vis absorption spectrophotometry [59,69,271] or via high-performance liquid chromatography HPLC [32,48,85], which are the most commonly used techniques for this purpose.

The percentage of drug in the organic solvent is then established and quantified by means of spectrophotometry or HPLC. This can be calculated as drug loading (DL) or entrapment efficiency (EE) according to the following equations:

(1) $$\mathrm{DL}\;(\%)=\frac{\mathrm{Drug}\;\mathrm{content}}{\mathrm{Weight}\;\mathrm{of}\;\mathrm{complex}}\times 100$$

(2) $$\mathrm{EE}\;(\%)=\frac{D_{T}-{\;D}_{S}}{D_{T}}\times 100;\;D_{T}=\mathrm{total}\;\mathrm{drug}\;\mathrm{amount},\;D_{S}=\mathrm{total}\;\mathrm{drug}\;\mathrm{determined}\;\mathrm{in}\;\mathrm{the}\;\mathrm{organic}\;\mathrm{solvent}$$

## 11. Conclusions

Cyclodextrins (CDs) are cyclic oligosaccharides that behave as natural cavitands that are biodegradable and lacking in toxicity, with a plethora of possibilities in pharmaceutical and food industry. Compartmentalization of isolated molecules in the cavities of cyclodextrins improves their stability by preventing undesired reactions at physiological pH and achieves an increase in the half-life of the active pharmaceutical ingredients, improving their distribution and therapeutic activity. CD complexation is also able to solve bioavailability issues of active pharmaceutical ingredients in certain pharmaceutical dosage forms, such as pediatric and ophthalmic preparations. The beneficial effects of cyclodextrins on the bioavailability of various nutraceuticals has been also considered. Cyclodextrin-related polymeric nanostructures have found broad application in the selective delivery of drug molecules to treat specific tissues. CD-pendent polymers can serve as hosts to encapsulate a large number of drug molecules that cannot be readily included via free cyclodextrins. The synthetic routes for the production of polymers based on cyclodextrins and their inclusion complexes with active principles have been reviewed. Taking into account that in order to verify the improvement in the bioavailability of drugs it is essential to have sensitive and selective analytical techniques that allow differentiating the included drugs from their free forms, the analytical techniques used in the characterization of inclusion complexes have been reviewed and summarized.

In today’s era of personalized medicine, the possibility to use natural, non-toxic cyclodextrins and their chemically modified and custom-designed derivatives, including polymeric nanomaterials, enables sustained delivery and improved therapeutic effects. In this context, the present review aims at providing readers with a critical perspective of recent developments in the use of cyclodextrins and related nanomaterials to improve the bioavailability of drug molecules and other bioactive compounds, including agrochemicals and nutraceuticals. We hope that the combination of these developments with synthetic and analytical aspects will stimulate the interest of researchers in the fascinating world of the supramolecular chemistry of cyclodextrins.

## Figures and Tables

**Table 1:** Physicochemical properties of natural cyclodextrins.

CD	Cavity Diameter (Å)	Outer Diameter (Å)	Cavity Volume (Å^3^)	Height (Å)	Molecular Weight (g/mol)	Aqueous Solubility (mg/mL)
α	4.7–5.3	14.6	174	7.9	973	145
β	6.0–6.5	15.4	262	7.9	1135	18.5
γ	7.5–8.3	17.5	427	7.9	1297	232

**Table 2:** Literature survey of the use of cyclodextrins for increased bioavailability of drugs and other bioactive compounds.

Drug	Bioactivity	Cyclodextrin/Polymer	Improvement/Enhancement	Ref.
Atorvastatin	Anti-hyperlipidemic drug that interferes in cholesterol biosynthesis	β-CD-based nanosponges cross-linked with carbonyl diimidazole	Improved solubilization and dissolution rate; enhanced oral bioavailability	[68]
Boswellic acids	Triterpenic acids with anti-inflammatory activity	Complexes with β-CD and HPβ-CD	Drug release and oral bioavailability enhancement due to the increase in the solubility	[30]
Camptothecin	Alkaloid with anticancer activity	HPβ-CD; 6-O-capro-β-CD; and polymeric nanoparticles of these CDs with poly-ε-caprolactone and poly (lactide-co-glycolide)	Enhanced anti-tumor activity due to the stabilization of the active form (lactone)	[52]
Camptothecin, Luotonin A	Alkaloids with anticancer activity	Complexes with β-CD and HPβ-CD	Enhanced solubility and stability. Improvement in anti-tumor activity with luotonin A reaching camptothecin-like activity	[50]
Carbamazepine	Treatment of epilepsy and neuropathic pain	Inclusion complexes with β-CD, HPβ-CD, and γ-CD	Improved equilibrium solubility, in vitro dissolution rates, and oral bioavailability	[69]
Curcumin	Natural hydrophobic polyphenolic compound with a variety of pharmacological activities	γ-CD complex	Increase in plasma levels of curcumin; significant increase in oral bioavailability	[70,71]
Cyclosporin	Peptide isolated from fungus acting as immunosuppressant and in ophthalmic preparations	Polymers based on HPβ-CD and poly(ethyleneglycol) diglycidyl ether	The polymer incorporating HPβ-CD allows good penetration of cyclosporine through sclera and ocular tissues	[72]
Doxorubicin	Anti-cancer drug	β-CD grafted onto graphene oxide with L-phenylalanine as a linker	Enhancement of 10–40% in cytotoxicity activity with regard to free drugs	[73]
Doxorubicin	Anti-cancer drug	Nanogels based on β-cyclodextrin-modified chitosan	Enhancement in the sustained release profile into the tumor cells; increased anti-tumor activity	[74]
Eugenol	Methoxyphenol derivative with analgesic, anti-inflammatory properties employed as cement component in clinical dentistry	Complexes with α-CD and β-CD	Suggested increased bioavailability	[75]
Flavonoids	Natural polyphenolic compounds with antioxidant and anti-inflammatory activity	Several CDs	Enhanced oral bioavailability with an increase in the biological activity	[76]
Flurbiprofen	Nonsteroidal anti-inflammatory drug employed in the treatment of intraocular inflammation	γ-CD-based polypseudorotaxane hydrogels	Improved the precorneal drug retention ability, corneal permeability, and intraocular bioavailability	[77]
Genistein	Isoflavone with anti-cancer activity	Amino-appended β-CDs	Complex solubility increased 1000-fold compared with free genistein; cytotoxic activity also increases	[78]
Ginsenoside Re	A triterpene present in ginseng root with effects on ischemic damage of the cardiovascular system	Complexes with α-CD, β-CD, and *γ*-CD	Dissolution rate is increased ca. 10% and bioavailability is enhanced by 1.71% for the complex with *γ*-CD with regard to the free compound	[79]
Irinotecan, Topotecan, Doxorubicin	Anticancer drugs and topoisomerase inhibitors	Heptakis-[6-deoxy-6-(3-sulfanyl HX acid)]-β-cyclodextrin; HX = acetic or propionic acid	pH-controlled release of anticancer drugs due to negative charge of polyanionic CDs	[80]
Melatonin/sulforaphane hybrid	Treatment of ischemic stroke	HPβ-CD complexes	A 34-fold increase in solubility; stabilization at several pH and temperature values	[81]
17-α-Methyltestosterone	Androgen	β-CD complex	A 6-fold hydrosolubility enhancement, with sustained release of the hormone	[82]
Methotrexate 5-Fluorouracil	Antimetabolites employed for cancer treatment	β-CD/glycerol organogel based on self-aggregation of β-CD	Sustained drug release providing an improved therapeutic effect	[83]
Morin	Flavonoid present in tea, wine, fruits, and vegetables acting as an antioxidant, anti-inflammatory	Complexes with HPβ-CD	Increased solubility and dissolution rate; oral bioavailability increased 4.2-fold compared with the free natural product	[84]
Nifedipine	Calcium-channel blocking agent for hypertension treatment	Multicomponent complexes with β-CD and aspartic acid	Enhancement of solubility and increase in dissolution rate	[85]
Nintedanib	Kinase inhibitor for the treatment of pulmonary fibrosis	Complexes with sulfobutyl ether-β-cyclodextrin	Improved stability at physiological pH and enhanced membrane transport	[86]
Norfloxacin	Fluoroquinolone antibiotic employed for urinary tract infections	β-Cyclodextrin-based nanosponges cross-linked with diphenyl carbonate	Controlled release of norfloxacin with enhanced membrane permeability and antibiotic efficiency	[87]
Oleuropein Hydroxytyrosol Tyrosol	Polyphenols present in olives and olive oil (functional foods) with antioxidant activity	Complexes with native β-CD	A 12–14% enhancement in the anti-oxidant activity (DPPH method)	[88]
Sulforaphane	Natural product isolated from broccoli with anticancer properties	Complexes with α-CD	Chemical stabilization	[89]
Tamoxifen	Antiestrogen—employed in hormone treatment of breast cancers	M-β-CD; HP-β-CD; and Sulfobutyl ether-β-cyclodextrin	Enhanced solubility and dissolution rate of tamoxifen inclusion complexes. Significant improvement in oral bioavailability	[90]
Umbelliferon	Coumarin derivative with lipid-lowering capability, and anticarcinogenic and HIV-inhibition activities	Complexes with α-CD	Potential sunscreen agent for protection from UV radiation	[91]

**Table 3:** Summary of some relevant CD inclusion complexes with indication of the analytical techniques employed for their analysis and characterization.

Guest	Host ^a^	Solution State			Solid State		Ref.
		Stoich.	*K*_ass_	Technique ^a^	Preparation	Technique	
Aceclofenac	HPβ-CD	1:1	222.11	HPLC ^b^	Kneading Coprecipitation	FTIR, DSC, PXRD	[235]
Adamantane– Erythropoietin	mPEG β-CD	1:1	NP	^1^H-NMR	Lyophilization	FTIR, DSC, TGA, PXRD, MALDI-TOF	[236]
Berberine Coptisine Palmatine Epiberberine Dehydrocorydaline	SBE-β-CD	1:1 1:1 1:1 1:1 1:1	19,200 ± 10^3^ 19,100 ± 1300 4700 ± 200 3000 ± 300 2500 ± 200	Fluorescence ^b^, UV/Vis, ITC			[237]
Betulin 3,28- diphthalate Betulin 3,28- disuccinate Betulin 3,28-disulfate	HPβ-CD	1:1 1:2 1:1 1:2 1:1 1:2	7.1 × 10^4^ 3.6 × 10^8^ 8.3 × 10^4^ 3.5 × 10^8^ 4.0 × 10^4^ 1.3 × 10^7^	ACE ^b^			[238]
Betulin 3,28- diphthalate Betulin 3,28- disuccinate Betulin 3,28-disulfate	DMβ-CD	1:1 1:2 1:1 1:2 1:1 1:2	9.5 × 10^4^ 3.3 × 10^7^ 1.8 × 10^5^ --- 9.3 × 10^4^ 1.7 × 10^8^	ACE ^b^			[239]
Betulin 3,28- diphthalate Betulin 3,28- disuccinate	HPγ-CD	1:1 1:1	1.7 × 10^7^ 1.3 × 10^7^	ACE ^b^			[240]
Bifonazole Clotrimazole Miconazole Tioconazole	β-CD SBEβ-CD β-CD SBβ-CD β-CD SBEβ-CD β-CD SBEβ-CD	1:1 1:1 1:1 1:1 1:1 1:1 1:1 1:1	2.5 × 10^3^ 5.2 × 10^4^ 4.5 × 10^2^ 2.9 × 10^3^ 5.0 × 10^2^ 1.5 × 10^3^ 1.5 × 10^3^ 9.3 × 10^3^	CD ^b^, UV/Vis			[235]
Bifonazole Miconazole	β-CD HPβ-CD β-CD HPβ-CD	1:1 1:1 1:1 1:1	2767 2487 932 1012	CE ^b^			[241]
Cefuroxime Axetil	β-CD β-CD-L-Arg	1:1 1:1	339.74 ± 1.5 498.98 ± 2.7	UV/Vis ^b^	Spray drying	DSC, PXRD, SEM	[242]
Celecoxib	SBEβ-CD	1:1	8131	UV/Vis ^b^, HPLC, ^1^H-NMR, H-H ROESY	Freeze drying		[243]
Chysin	HPβ-CD	1:1	1090	UV/Vis ^b^ ^1^H-NMR	Kneading Coprecipitation	FTIR, TGA, SEM	[244]
Curcumin	β-CD β-CD NS4 β-CD NS6 β-CD NS8	1:1 1:1 1:1 1:1	487.34 4972.90 4167.50 3567.87	HPLC ^b^	Freeze drying	FTIR, DSC, PXRD	[245]
Diacerhein	HPβ-CD	1:1 1:1	45 * 22 ^#^	UV/Vis ^b,^* HPLC ^b,#^	Kneading Coevaporation Freeze drying	FTIR, DSC	[246]
Eprosartan mesylate	β-CD	1:1	280.78	UV/Vis ^b^ ^1^H-NMR	Microwave irradiation	FTIR, DSC, PXRD, SEM	[247]
Estradiol	β-CD HPβ-CD	1:1 1:1	3.14 × 10^4^ 3.22 × 10^4^	Fluorescence ^b^ UV/Vis, CD			[248]
Erlotinib	α-CD β-CD γ-CD HPβ-CD RAMEB CMβ-CD SBβ-CD	1:1 1:1 1:1 1:1 1:1 1:1 1:1	142 ± 9 * 268 ± 11 * 324 ^#^ 54 ± 4 * 235 ± 10 * 434 ± 13 * 1396 ± 66 * 2380 ± 62 *	ACE ^b,^*, LC-MS ^b,#^, ^1^H NMR, H-H ROESY, UV, ESI-MS	Co-evaporation, kneading, vacuum desiccation, coprecipitation	XRD	[249]
Etodolac	HPβ-CD HPβ-CD-L-Arg	1:1 1:1	162.11 ± 6.24 2573.31 ± 11.31	UV/Vis ^b^, ^1^H NMR	Coevaporation Spray drying	FTIR, DSC, PXRD	[250]
Flurbiprofen	β-CD	1:1	2483.8	^1^H NMR *, H-H ROESY			[251]
Folic Acid	β-CD	1:1 1:2	944 ± 79 * 159 ± 26 ^#^	TDA-CE * ACE^#^			[252]
Genistein	Ab-CD_1_ Aβ-CD_2_ Aβ-CD_3_ Aβ-CD_4_	1:1 1:1 1:1 1:1	31,300 15,692 35,386 20,745	UV/Vis ^b^, ^1^H-NMR, H-H ROESY	Lyophilization	FTIR, PXRD, SEM	[79]
Genistein Daidzein	SBEβ-CD	1:1 1:1	34,926 ± 4500 13,131 ± 980	UV/Vis ^b^	Kneading	ATR-FTIR	[253]
Gemfibrozil	β-CD HPβ-CD Meβ-CD	1:1 1:1 1:1 1:2	760 ± 30 * 530 ± 20 * 440 ± 20 *	Fluorescence ^b,^* ^1^H NMR ^b^, UV ^b^	Co-evaporation, kneading, vacuum desiccation, coprecipitation		[254]
Ginsenoside Re	α-CD β-CD γ-CD	1:1 1:1 1:1	22 612 1.4 × 10^4^	HPLC ^b^	Lyophilization	FTIR, DSC, PXRD	[80]
Guanosine	β-CD	1:1 1:1	59.79 * 151.97^#^	UV/Vis ^b,^* Fluorescence ^b.# 1^H-NMR, ^13^C-NMR	Coprecipitation	FTIR, DSC, TGA, PXRD, FESEM	[255]
Guanosine	β-CD HPβ-CD SBEβ-CD	1:1 1:1 1:1	87.53 *, 70.79 ^#^ 91.61 *, 98.97 ^#^ 103.29 *, 190.76 ^#^	UV/Vis ^b,^* Fluorescence ^b,#^	Coprecipitation	FTIR, DSC, TGA, PXRD, FESEM	[256]
Harmane Harmine	β-CD DMβ-CD TMβ-CD HPβ-CD β-CD DMβ-CD TMβ-CD HPβ-CD	1:1 + 1:2 1:1 + 1:2	345 ± 42 207 ± 50 121 ± 29 602 ± 46 201 ± 14 148 ± 27 195 ± 14 353 ± 46	Fluorescence ^b^ UV/Vis			14
Hinokitiol	α-CD β-CD	1:2 1:1	175 187	UV/Vis ^b^, H-H ROESY	Grinding	FTIR, DSC, PXRD, Fluorescence	[257]
Inosine	β-CD	1:1 1:1	33.59 * 104.53 ^#^	UV/Vis ^b,^* Fluorescence ^b,#^	Kneading Coevaporation	FTIR, DSC; XRD, SEM	[258]
*(R)*-Ketoprofen *(S)*-Ketoprofen	β-CD	1:1 1:1	2750 *, 4088 ^#^ 1299 *, 2547 ^#^	UV/Vis ^b,^* Fluorescence ^b,#^	Coevaporation	RAMAN	[259]
Myrtenol	β-CD	1:1	--	^1^H-NMR, H-H ROESY	Kneading Slurry	DSC, TGA, PXRD, SEM	[200]
Nifedipine	β-CD β-CD:Asp	1:1 1:1	99 ± 2 117 ± 4	HPLC ^b^, ^1^H-NMR	Kneading	FTIR, DCS, TGA, PXRD, SEM	[86]
Nifurtimox	β-CD SBEβ-CD	1:1 1:1	236.79 359.57	UV/Vis ^b^, ^1^H-NMR, ^13^C-NMR	Coevaporation	FTIR, DSC, TGA, PXRD, SEM	[260]
Olanzapine	β-CD DMβ-CD	1:1 1:1	100 ± 3 278 ± 5	UV/Vis ^b^, ^1^H-NMR	Kneading	FTIR	[261]
4-Phenylbutyrate	α-CD β-CD γ-CD	1:1 1:1 2:1	481 ± 26 178 ± 23 119 ± 9	HPLC ^b^, CD, ^1^H-NMR, H-H COSY, H-H ROESY			[262]
PNU 2FPNU 2MPNU 2MOPNU	β-CD	1:1 1:1 1:1 1:1	1316 600 2200 5071	Fluorescence ^b^			[263]
20*S*-Protopanaxatriol	EDBA-bis-β-CD	1:1	995.94 ± 0.07	UV/Vis ^b^, ^1^H NMR, H-H ROESY	Coprecipitation	FTIR, PXRD, SEM	[264]
6-Propyl-2-thiouracil	α-CD	1:1	3297.57 ± 0.15	UV/Vis ^b^, ^1^H-NMR	Coevaporation	FTIR, DSC, TGA, PXRD, SEM	[265]
Sulfabenzamide	β-CD Mβ-CD	1:1 1:1	2.4 × 10^5^ 2.2 × 10^4^	UV/Vis ^b^, ^1^H-NMR	Lyophilization	FTIR, PXRD, SEM	[266]
Sulfisoxazole Sulfamethizole Sulfamethazine	β-CD	1:1 1:1 1:1	650 1532 714	UV/Vis ^b^ ^1^H-NMR	Coevaporation	DSC	[267]
Temoporfin	β-CD NS cmβ-CD NS	1:1 1:1	6.3 × 10^6 ^ 1.2 × 10^6^	Fluorescence ^b^			[268]
Tenofovir	β-CD	1:1	863 ± 32	UV/Vis ^b^	Coprecipitation	FTIR, DSC, TGA, PXRD, SEM	[269]
Trioxaadamantane Adamantoid scaffolds: G2 G3 G4	β-CD	1:1 1:1 1:1 1:1	969 ± 62 173 ± 11 2990 ± 65 4140 ± 193	ITC ^b^, ^1^H-NMR	Cocrystallization	PXRD	[270]

^a^ Selected abbreviations: **Aβ-CD**: amino-β-cyclodextrin; **ACE**: affinity capillary electrophoresis; **β-CD:Asp**: β-cyclodextrin:aspartic acid; **β-CD-LArg** β-CD-L-arginine; **cmβ-CD**: carboxymethyl-β-cyclodextrin. **EDBA-bis-β-CD**: bridged-bis-[6-(3,3′-(ethylenedioxy) bis (propylamine))-6-deoxy-β-CD]; **FESEM**: field emission scanning electron microscope; **2FPNU**: 1-(2-chloroethyl)-3-(2-fluorophenyl)-1-nitrosourea; **HPβ-CD-LArg**: hydroxypropyl-β-cyclodextrin-L arginine; **Mβ-CD**: methyl-β-cyclodextrin; **mPEGβ-CD**: monoPEGylated β-cyclodextrin; **2MOPNU**: 1-(2-chloroethyl)-3-(2-methoxyphenyl)-1-nitrosourea; **2MPNU**: 1-(2-chloroethyl)-3-(2-methylphenyl)-1-nitrosourea; **NS**: nanosponge; **PNU**: 1-(2-chloroethyl)-3-phenyl-1-nitrosourea; **SBEβ-CD**: sulfobutylether-β-cyclodextrin; **TDA-CE**: Capillary electrophoresis based on Taylor dispersion analysis. ^b^ Analytical technique used in the *K*_ass_ calculation. The * and ^#^ symbols are employed to associate the numeric *K*_ass_ value with the technique employed for its determination.

## Data Availability

Not applicable.
